# Novel Unsymmetric 3,5-Bis(benzylidene)-4-piperidones That Display Tumor-Selective Toxicity

**DOI:** 10.3390/molecules27196718

**Published:** 2022-10-09

**Authors:** Aruna Chhikara, Praveen K. Roayapalley, Hiroshi Sakagami, Shigeru Amano, Keitaro Satoh, Yoshihiro Uesawa, Umashankar Das, Swagatika Das, Edgar A. Borrego, Cristina D. Guerena, Clare R. Hernandez, Renato J. Aguilera, Jonathan R. Dimmock

**Affiliations:** 1 Department of Chemistry, Dyal Singh College, University of Delhi, New Delhi 110003, India; 2 Drug Discovery and Development Research Cluster, College of Pharmacy and Nutrition, University of Saskatchewan, Saskatoon, SK S7N 5E5, Canada; 3 School of Dentistry, Meikai University, Sakado 350-0283, Japan; 4 Department of Medical Molecular Informatics, Meiji Pharmaceutical University, Tokyo 204-8588, Japan; 5 Department of Biological Sciences and Border Biomedical Research Center, The University of Texas at El Paso, El Paso, TX 79968-0519, USA

**Keywords:** conjugated unsaturated ketones, cytotoxicity, 4-piperidones, selectivity index, apoptosis, reactive oxygen species, mitochondrial membrane potential

## Abstract

Two series of novel unsymmetrical 3,5-bis(benzylidene)-4 piperidones **2a**–**f** and **3a**–**e** were designed as candidate antineoplastic agents. These compounds display potent cytotoxicity towards two colon cancers, as well as several oral squamous cell carcinomas. These compounds are less toxic to various non-malignant cells giving rise to large selectivity index (SI) figures. Many of the compounds are also cytotoxic towards CEM lymphoma and HL-60 leukemia cells. Representative compounds induced apoptotic cell death characterized by caspase-3 activation and subG1 accumulation in some OSCC cells, as well as the depolarization of the mitochondrial membrane potential in CEM cells. A further line of inquiry was directed to finding if the SI values are correlated with the atomic charges on the olefinic carbon atoms. The potential of these compounds as antineoplastic agents was enhanced by an ADME (absorption, distribution, metabolism, and excretion) evaluation of five lead molecules, which revealed no violations.

## 1. Introduction

The emphasis in our laboratories is the creation of novel cytotoxins. These compounds are conjugated unsaturated ketones that react with thiols but less readily with the amino and hydroxy groups [1,2,3] that are found in nucleic acids. Hence, genotoxicity may be absent in these compounds.

The objectives of the present investigation are threefold. First, the design, syntheses and cytotoxic evaluation of two series of novel enones **2a**–**f** and **3a**–**e** was planned. These compounds were intended to be more toxic to neoplasms than to non-malignant cells. Second, an effort was made to find some of the ways by which cytotoxicity is mediated. Third, a decision was made to evaluate a theory that selective toxicity values are related to the differences between the atomic charges on the olefinic carbon atoms.

**Figure 1:**
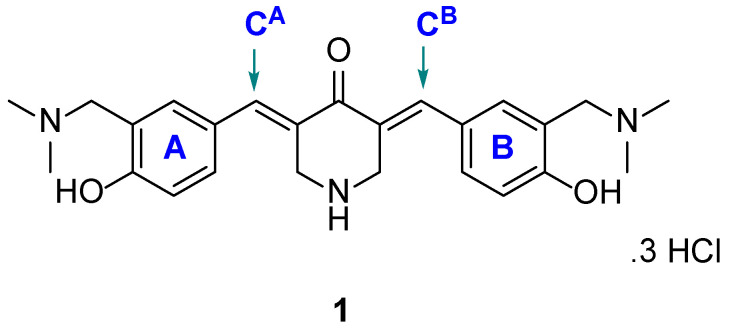
Structure of **1**.

Several years ago, the 3,5-bis(benzylidene)-4-piperidone **1** (Figure 1) was shown to display significant cytotoxic properties towards several neoplastic cell lines [4,5]. The two aryl substituents were chosen to form hydrogen bonds with cellular constituents. In addition, the hydroxyl group will increase the hydrophilicity of the molecules, while the deamination of **1** could occur to produce a chemically reactive species. However, while this compound was cytotoxic to a number of malignant cell lines, it has low selectivity index (SI) values (CC_50_ figure towards on-malignant cell lines / CC_50_ value towards a neoplastic cell line) of 3–7 [4,5].

**Figure 2:**
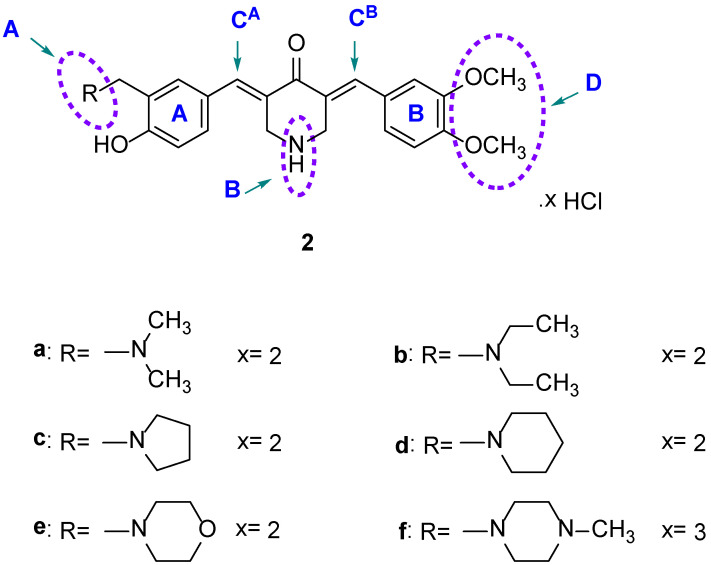
Structures of **2a**–**f**.

**Figure 3:**
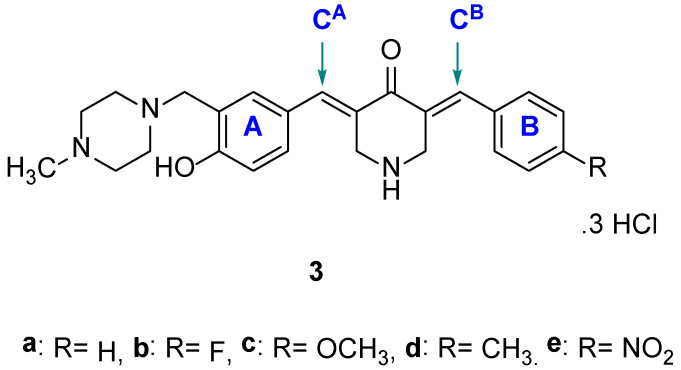
Structures of **3a**–**e**.

The design of analogs of **1** was based on the following considerations. (i) One of the rings contained a dimethylaminomethyl and a 4-hydroxy group that are present in **1** to give **2a**. (ii) The basicity and the size of the dimethylamino group in **2a** may affect cytotoxic properties, so **2b**–**f** were designed to explore this possibility (Figure 2). (iii) The 1,5-diaryl-3-oxo-1,4-pentadienyl group was mounted on a piperidine ring, so that, if the development of this series of compounds was warranted, various structural modifications on the piperidyl nitrogen atom could occur. (iv) A number of studies revealed that the conjugated arylidene ketones, which have methoxy groups on the aryl rings, are potent cytotoxins [6,7,8]. Consequently, this substituent was incorporated into the aryl ring of series **2**. Series **3** was designed so that groups having different electronic properties were placed in ring B (Figure 3). In this way, the atomic charges on the olefinic carbon atoms varied, which may correlate with cytotoxic potencies, *vide infra.*

In summary, the present investigation aims to prepare two novel series of compounds and investigate whether cytotoxic potencies and tumor-selective toxicity are displayed. If these criteria were achieved, the modes of action of representative compounds would be investigated and the theory explored that SI values are related to differences in the atomic charges on the olefinic carbon atoms.

## 2. Results 

**Figure 4:**
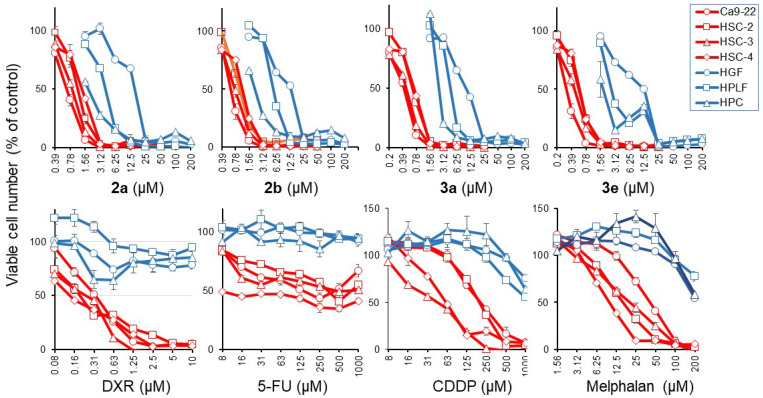
Dose–response curve of the cytotoxicity of **2a**, **2b**, **3a**, **3e**, and anticancer drugs. Cells were incubated for 48 h without (control) or the indicated concentrations of test samples, and the viable cell number was determined by the MTT method and expressed as % of control. Each value is represented as the mean ± S.D. of triplicate determinations.

**Scheme 1:**
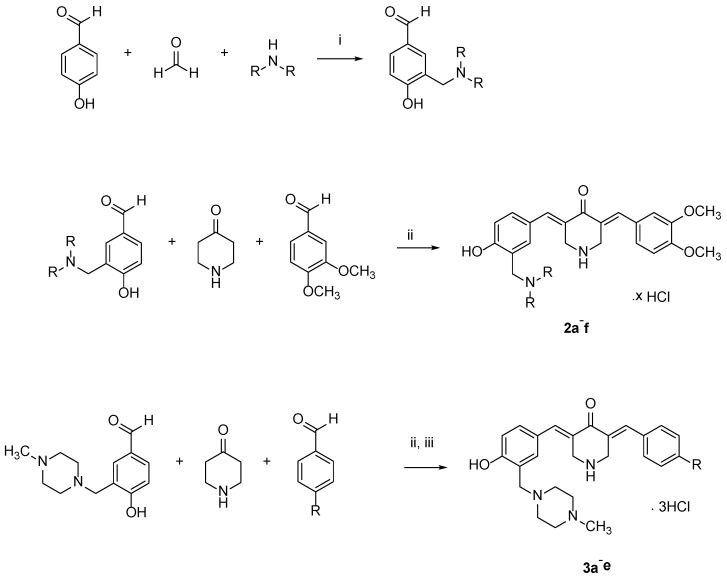
Synthesis of **2a**–**f** and **3a**–**e**. i = CH_3_OH / HCl / K_2_CO_3_; ii = CH_3_COOH /HCl/ K_2_CO_3_; iii = CH_3_COOH/ HCl /K_2_CO_3_.

The compounds in series **2** and **3** were synthesized by the procedure outlined in Scheme 1. All of the compounds in series **2** and **3**, as well as **1**, were examined against human HCT116 and HT29 colon cancer cells, as well as human CRL1790 non-malignant colon cells. These results are presented in Table 1. In addition, **2a**–**f** and **3a**–**e** were screened against human Ca9-22, HSC-2, HSC-3, and HSC-4 squamous cell carcinomas; these data are portrayed in Table 2. The compounds were also evaluated against HGF, HPLF, and HPC non-malignant cells, and these results are indicated in Table 3. Dose–response cytotoxicity induction by all and representative compounds are shown in the supplementary section (Figure S1 and Figure 4), respectively. Representative compounds were assessed against human CEM lymphoma and human HL60 promyelocytic leukemia cells (Table 4).

**Figure 5:**
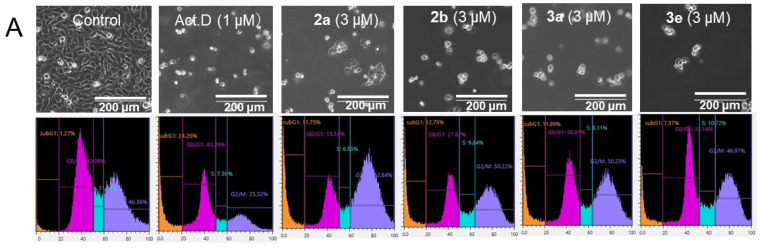

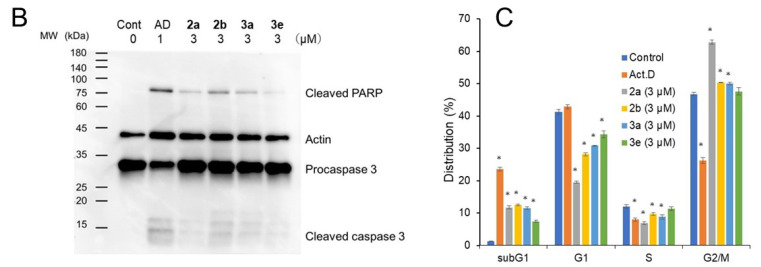
Induction of cell shrinkage (**A**), caspase 3 activation (**B**), and subG1 and G2/M accumulation by **2a**, **2b**, **3a**, and **3e**. Ca9-22 cells were treated for 24 h with vehicle (control), actinomycin D (Act.D) (1 μM), **2a**, **2b**, **3a**, and **3e** (3 μM) in Ca9-22 cells. Each value is represented as the mean ± S.D. of triplicate determinations. * *p* < 0.05 vs. control (Bonferroni’s post-test) (**C**).

**Figure 6:**
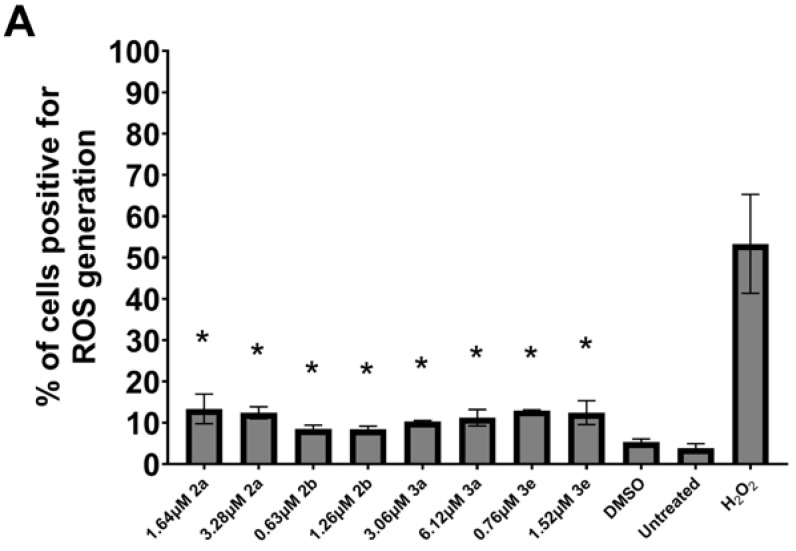

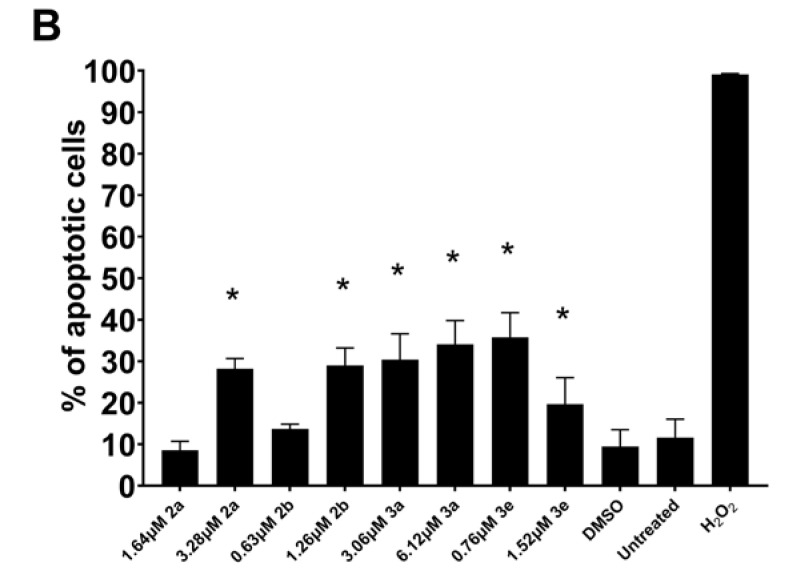

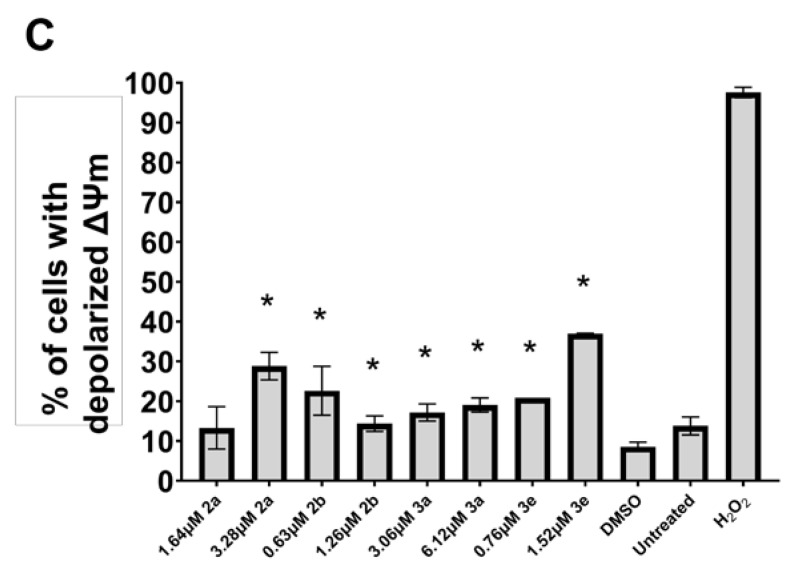
Induction of ROS generation (**A**), apoptosis (**B**), and the depolarization of mitochondrial membrane potential (**C**) by **2a**,**b** and **3a**,**e** in CEM cells. * *p* < 0.01 compared to DMSO for (**A**), * *p* < 0.05 compared to DMSO for (**B**,**C**).

Some mode-of-action studies were undertaken as follows. The induction of cell shrinkage (A), caspase (B), and the accumulation of G2/M and subG1 of the cell cycle in Ca9-22 cells are shown in Figure 5. **2a**, **2b**, **3a**, and **3e** increased the concentration of ROS (A) and induced apoptosis (B) and the depolarization of the mitochondrial membrane potential (C) (Figure 6) in CEM cells. The atomic charges on the olefinic carbon atoms of **1**, **2a**–**f**, and **3a**–**e** are presented in Supplemental Table S1. The correlations and trends to a correlation of these atomic charges with the SI values of **2a**–**f**, and **3a**–**e** are presented in Table 5. Some of the physicochemical properties of various lead molecules were obtained and are listed in Table 6.

## 3. Discussion

The first challenge to overcome was the synthesis of the compounds in series **2** and **3**. Previous studies in our laboratories with cyclic ketones and aryl aldehydes gave products with one or two arylidene groups on the alicyclic ring depending on the molecular ratios of the reactants [9,10,11,12]. However, the reaction of 4-piperidone with an aryl aldehyde gave only the 3,5-bis(benzylidene)-4-piperidone. Hence, in order to prepare the compounds in series **2** and **3**, a mixture of the appropriate Mannich base, aryl aldehyde and 4-piperidone was heated together, and the desired products were obtained by column chromatography [13,14,15].

An initial bioevaluation was undertaken to determine whether the compounds in series **2** and **3** have cytotoxic properties. A major interest in our laboratories is searching for novel compounds that inhibit the growth of human colon cancer cells [16]. Hence, an initial assessment of **2a**–**f** and **3a**–**e** was undertaken using human HCT116 and HT29 cancer cells, and the results are presented in Table 1. 

All of the compounds in series **2** and **3** have IC_50_ values below 4 µM and are potent cytotoxins towards HCT116 and HT29 cells. In particular, the IC_50_ values of **2b**, **d**, and **f** and **3a**, **b**, and **e** are below 1 µM towards both HCT116 and HT29 cells. Hence, one should consider incorporating diethylamino, piperidino, and 4-methyl-1-piperazinyl groups into the design of future analogs of series **2**. In the case of series **3**, electron-attracting substituents should be placed in ring B.

5-Flurouracil (5-FU) is used in treating colon cancers [17]. All compounds in series **2** and **3** are more potent than 5-FU to both HCT116 and HT29 cells except **2c**, **e**, and **3d** are equitoxic with 5-FU towards HCT116 cells.

With the encouraging results to hand, the next step was to find if the compounds in series **2** and **3** are more toxic to various neoplasms than to a non-malignant cell line. Hence, the compounds were evaluated against human CRL1790 non-malignant colon cells, and the results are presented in Table 1. From these data, one may calculate the selectivity index (SI) values, which are the ratios of the CC_50_ figures of a compound towards CRL1790 cells and the IC_50_ values against HCT116 or HT29 cells. The SI figures are presented in Table 1. Compounds with SI figures over 50 are **2b**, **d**, **3a** (HCT116 cells) and **2b**, and **3a** (HT29 cells), which serve as lead molecules for analog development. All of the SI values for the compounds in series **2** and **3** towards HCT116 cells are greater than the figures for 5-FU. Similarly, these compounds have higher SI values than 5-FU when the HT29 screen is concerned.

Two important properties of candidate cytotoxic agents are their antineoplastic potencies and greater toxicity towards neoplasms than non-malignant cells. Hence potency–selectivity expression (PSE) values were calculated, which are the reciprocal of the average potency towards neoplastic cells multiplied by the average SI values times 100. These figures are presented in Table 1 and indicate that **2b** and **3a**, **b**, and **e** have high values and serve as lead molecules. The PSE figures for all of the compounds in series **2** and **3** are greater than the value for 5-FU. In general, series **2** and **3** have higher SI and PSE values than **1**.

The potential utility of this novel cluster of compounds will be enhanced if other groups of tumors are also susceptible to these molecules. Consequently, **2a**–**f** and **3a**–**c** were evaluated against human Ca9-22, HSC-2, HSC-3, and HSC-4 squamous cell carcinomas. The results are presented in Table 2. All compounds showed potent cytotoxicity rather than cytostatic growth inhibition (Figure S1 and Figure 4).

The average CC_50_ values calculated from the dose–response curve of OSCC cells (indicated in red in Figure 4) in Table 2 reveal that, in general, the compounds in series **2** and **3** are potent cytotoxins. In particular **2b**, **d** and **3a**, **b**, **e** have average CC_50_ values of less than 1 µM and may be considered lead molecules. On the other hand, **2e** has a very low average potency. This compound has a morpholinyl group, which possesses a much lower pKa than the basic groups found in **2a**–**d** for example [18]. In the future, the preparation of analogs of **2a**–**e** possessing groups of varying basicity should be prepared to determine if cytotoxic potency is proportional to the magnitude of the pKa values.

Comparisons were made between the average potencies of the compounds in series **2** and **3** with four reference drugs, namely 5-FU (an antimetabolite), melphalan (an alkylating agent; series **2** and **3** are considered to be thiol alkylators), CDDP (cisplatin, a DNA toxin), and doxorubicin (an anticancer antibiotic). Except for **2e**, the compounds in series **2** and **3** have average CC_50_ values lower than 5-FU, melphalan, and CDDP, while doxorubicin is more potent than **2a**–**f** and **3a**–**e**.

The next issue to be addressed is whether the compounds in series **2** and **3** are well tolerated by various non-malignant cells. Hence, **2a**–**f** and **3a**–**e** were evaluated against human HGF, HPLF, and HPC non-malignant cells, and the data are presented in Table 3. The compounds that cause the least toxicity to normal cells [average CC_50_ values, determined from the dose–response curves of normal oral cells (indicated by the blue color in Figure 4)] towards HGF, HPLF, and HPC cells in parentheses) are **2e** (>200 µM), **2c** (53.6 µM), **2f** (17.1 µM), **3a** (10.2 µM), and **3b** (10.1 µM). The structural features of these compounds should be considered when developing this series of compounds.

The SI figures of **2a**–**f** and **3a**–**e** towards Ca9-22, HSC-2, HSC-3, and HSC-4 were obtained by dividing the average CC_50_ values towards HGF, HPLF, and HPC non-malignant cells (Table 3) by the CC_50_ value of a compound towards a specific neoplasm (Table 2). The SI values are presented in Table 2. Compounds with the highest SI values are **3a**, **b**, and **e**, which have average SI values greater than 10. The PSE figures of **2a**–**f** and **3a**–**e** listed in Table 3 reveal that the compounds with the highest figures are **2d**, **3a**, **b**, and **e**, with PSE values in excess of 1000.

In Ca9-22 cells, **2a**, **b**, **3a**, and **e** induced cell shrinkage (A), caspase 3 activation (assessed by the cleavage of PARP and procaspase) (B), and accumulation at the subG1 population following G2/M accumulation (C) (Figure 5). A dose–response study demonstrated that the optimal concentration of **2b** for the subG1 population was 2.5 μM (Supplementary Figure S2). The bell shape of the stimulation curve may be due to the activation of apoptosis-related enzymes such as caspase 3 and caspase-activated DNase(s). However, it should be noted that the extent of apoptosis induced by these compounds (assessed by either caspase activation or the accumulation of the subG1 population) was only half that induced by actinomycin D, a positive control of apoptosis. This may reflect the difference of action points: **2b** reproducibly induced the accumulation of G2/M before subG1 accumulation, while the latter did not but rather inhibited G2/M accumulation (Figure 4 and Supplemental Figure S2).

Hence, various members of series **2** and **3** were evaluated against human CEM acute lymphoblastic lymphoma cells and human HL-60 promyelocytic leukemia cells. The biodata generated is presented in Table 4.

The compounds selected, namely **2a**–**c**, **f**, **3a**, **b**, and **e**, were incubated for 24 h. However, since this time may be inadequate to cause significant toxicity to the malignant cells, the determinations were also carried out for 48 h. In general, greater toxicity was displayed after 48 h of incubation. The following comments apply to the results from 48 h of incubation.

The biodata in Table 4 for the CEM bioassay reveals that the most potent compound is **3b**, followed by **2a**, **b**, and **3e**. On the other hand, the weakest potency is displayed by **2c**, which, in general, is a similar observation to the antineoplastic properties recorded in Table 1 and Table 2.

In general, the compounds are less potent towards HL-60 cells than towards CEM lymphomas. The analogs with the lowest CC_50_ values towards HL-60 cells are **2a**, **f**, **3a**, and **b**, which serve as lead molecules.

An investigation was conducted to find some of the ways in which cytotoxicity was caused by the compounds in series **2** and **3**. Four representative compounds were chosen, namely **2a**, **b**, **3a**, and **e**, which are toxic to CEM cells (Table 4).

Using the CC_50_ and twice the CC_50_ concentrations of **2a**, **b**, and **3a**, **e**, the following observations were made. One way that compounds cause cellular damage is by increasing the concentration of reactive oxygen species (ROS) [19]. The results presented in Figure 6A reveal that **2a**, **b** and **3a**, **e** generate ROS, although this effect is likely only a minor contributor to cytotoxicity. Another way in which the compounds in series **2** and **3** exert their cytotoxicity is by causing apoptosis. The results presented in Figure 6B reveal that approximately 30% of the cells are apoptotic, and therefore this effect contributes significantly to the cytotoxicity of these compounds. A further biochemical mechanism that may contribute to the cytotoxicity of these compounds in series **2** and **3** is by causing the depolarization of the mitochondrial membrane potential (MMP). The data presented in Figure 6C reveal that, for **2a** and **3e**, this is a noticeable contributor to toxicity.

The atomic charges on the olefinic carbon atoms of **1**, **2a**–**f**, and **3a**–**e** were determined using Spartan’18 by equilibrium geometry, a basis set of 6-31G*, and the ωB97X-D methodology [20]. From this source, electrostatic, Mulliken, and natural charges were obtained and are presented in Table S1 in the supplemental portion of this report. The hypothesis that the SI figures are related to differences between the atomic charges on the C^A^ and C^B^ atoms was evaluated. Linear, semilogarithmic, and logarithmic plots were constructed between the average SI values of **1**, **2a**–**f**, **3a**–**e**, and Δv. These values are listed in Table S1. This method was repeated with the Mulliken charges and then with the natural charges. These procedures were repeated using the average SI values of **2a**–**f** and **3a**–**e** listed in Table 2.

Plots were made between the Δv and the SI data. All of the plots are positive, and 39% of the results generated are either significant (*p* < 0.05) or trends to a correlation (*p* < 0.10). Hence, some support is generated for the hypothesis that, as the differential between the atomic charges on the olefinic carbon atoms are elevated, so the selective toxicity rises.

The results generated for the compounds in series **2** and **3** are encouraging in terms of potency and selectivity. However, lead molecules should have appropriate physicochemical properties in order to pursue development. Several guidelines have been formulated, such as those originating from Lipinski et al. [21] and Veber and coworkers [22]. Five of the lead compounds, namely **2b**, **d** and **3a**–**c** were evaluated for some of their ADME characteristics, and the results are given in Table 6, which reveals that there are no violations. Hence, these data encourage the pursuit of the development of these compounds.

We have recently reported that 7-methoxy-3-[(1E)-2-phenylethenyl]-4H-1-benzopyran-4-one and 3-[(1E)-2-(4-hydroxyphenyl)ethenyl]-7-methoxy-4H-1-benzopyran-4-one showed higher tumor specificity against human oral squamous carcinoma (SI = 301 and 182, respectively), showing much lower keratinocyte toxicity than doxorubicin and 5-FU. Surprisingly, these two 3-styrylchromone derivatives induced the accumulation of G2/M phase cells and subsequent subG1 accumulation [23,24], in a similar way as **2a**, **2b**, and **3a** (Figure 5C). This suggests new research areas to be pursued.

## 4. Materials and Methods

### 4.1. Syntheses of Compounds

A Bruker Avance AMX 500 FT spectrometer (Billerica, MA, USA) equipped with a broad band observe (BBO) probe was used to obtain ^1^H and ^13^C nuclear magnetic resonance (NMR) spectra of compounds in deuterated chloroform (CDCl_3_) and deuterated dimethyl sulfoxide (DMSO-d_6_). Mass spectra were obtained using a Jeol JMS-T100 GCv Accu tof-gcV4G instrument (Peabody, MA, USA). A DigiMelt-MPA 160 instrument (Sunnyvale, CA, USA) was used to determine melting points that are uncorrected.

The intermediate 3-aminomethyl-4-hydroxybenzaldehydes were prepared by a general method. A solution of 4-hydroxybenzaldehyde (0.05 mol) in methanol (50 mL was added to a preheated mixture of paraformaldehyde (0.06 mol) and the appropriate amine (0.05 mol) in methanol (50 mL) at 70 °C for 30 min. After the addition of 4-hydroxybenzaldehyde, the mixture was stirred at 70 °C for 2 h. On completion of the reaction, the solvent was removed, and the residue was dissolved in diethyl ether. Hydrogen chloride gas was passed through the solution for 10 min to produce the hydrochloride salt, which precipitated. The solid formed was collected by filtration and washed thoroughly with ether and dried. The ^1^H NMR, ^13^C NMR and Mass spectra are consistent with the proposed structures. The spectra of the available compounds are presented in the Supplemental section (Spectra S1, Spectra S2 and Spectra S3).

The hydrochloride salts were treated with an aqueous potassium carbonate solution (10% *w*/*v*), and the mixture was extracted with chloroform. The removal of the organic solvent in vacuo afforded the free bases that were used in the syntheses of **2a**–**f** and **3a**–**e**.

The compounds in series **2** were prepared as follows. 3,4-Dimethoxybenzaldehyde (0.0025 mol) and the appropriate 3-aminomethyl-4-hydroxybenzaldehyde (0.0025 mol) were added to a suspension of 4-piperidone hydrochloride monohydrate (0.005 mol) in acetic acid (35 mL). Dry hydrogen chloride was passed through this mixture for 0.5 h. The mixture was stirred at room temperature for 24 h, and the precipitate that formed was collected, washed with water, and added to an aqueous solution of potassium carbonate (25% *w*/*v*, 25 mL). The resultant mixture was stirred for 0.5 h. The free bases were washed with water (50 mL) and dried. The compounds were purified by recrystallization from 95% ethanol and then by using a chromatography column of silica gel 60 and an eluting solvent of a mixture of methanol and chloroform (1:99). Hydrogen chloride gas was passed into a solution of the pure compound, which was dissolved in chloroform. The hydrochloride salts were collected, washed with acetone, and dried. The compounds in series **3** were prepared in the same manner as used in the syntheses of **2a**–**f**. The melting points of **2a**–**f** and **3a**–**e** are over 300 °C.

**(2a) 3-(3,4-Dimethoxybenzylidene)-5-(3-((dimethylamino)methyl)-4-hydroxybenzylidene)-4-piperidone dihydrochloride**

Yield: 40%. *^1^H NMR (DMSO-d_6_)*: δ 2.39 (s, 6H, 2× NCH_3_), 3.74 (s, 2H, Ar-CH_2_-N), 3.96 (s, 3H, -OCH_3_), 3.98 (s, 3H, -OCH_3_), 4.12 (s, 2H, NCH_2_), 4.14 (s, 2H, NCH_2_), 6.75 (s, 4H, Ar-H), 6.97 (d, 2H, Ar-H), 7.75 (s, 2H, 2× =CH). *^13^C NMR (DMSO-d_6_)*: δ 40.55, 53.56, 56.12, 114.84, 126.92, 139.75, 149.15, 151.06, 182.44. *HRMS (ESI): (m/z)*: 408.2057 (M^+^), Calc: 408.2049 for C_24_H_28_N_2_O_4_, 409.2087 (M + 1), 410.2130 (M + 2)

**(2b) 3-(3-Diethylaminomethyl-4-hydroxybenzylidene)-5-(3,4-dimethoxybenzylidene)-4-piperidone dihydrochloride**

Yield: 40%. *^1^H NMR (DMSO-d_6_)*: δ 0.95 (t, 6H, 2× N-CH_2_-CH**_3_**), 2.68 (q, 4H, 2× -CH**_2_**-CH_3_), 3.70 (s, 2H, Ar-CH_2_-N), 3.90 (s, 3H, -OCH_3_), 3.94 (s, 3H, -OCH_3_), 4.2 (d, 2H, -NCH2), 6.88–6.95 (m, 2H, Ar-H), 7.15 (s, 4H, Ar-H), 7.78 (s, 2H, 2× =CH). *^13^C NMR (DMSO-d_6_)*: 40.38, 46.65, 49.59, 56.14, 112.23, 114.84, 116.73, 117.84, 124.72, 126.14, 126.91, 139.76, 149.15, 159.17, 182.44. *HRMS (ESI): (m/z)*: 436.2304 (M^+^), Calc: 436.2362 for C_26_H_32_N_2_O_4_, 437.2338 (M + 1), 438.2378 (M + 2)

**(2c) 3-[3-(1-Pyrrolidinylmethyl)-4-hydroxybenzylidene]-5-(3,4-dimethoxybenzylidene)-4-piperidone dihydrochloride**

Yield: 50%. ^1^H NMR (DMSO-d_6_): δ 1.85 (br. s, 4H, 2× N-CH_2_-CH**_2_**), 2.65 (br. s, 4H, 2× N-CH**_2_**-CH_2_), 3.92 (br. s, 4H, 2× NCH2), 3.95 (s, 3H, -OCH_3_)_,_ 3.97 (s, 3H, -OCH_3_)_,_ 4.17 (s, 2H, Ar-CH_2_-N), 6.88–7.15 (m, 4H, Ar-H), 7.28 (s, 2H, Ar-H), 7.75 (s, 2H, 2 × =CH). *^13^C NMR (DMSO-d_6_)*: 23.08, 44.54, 51.54, 56.14, 112.23, 114.83, 124.72, 126.14, 126.91, 139.77, 149.15, 158.82, 182.45. *HRMS (ESI): (m/z)*: 434.2215 (M^+^), Calc: 434.2205 for C_26_H_30_N_2_O_4_ 435.2256 (M + 1), 436.2300 (M + 2)

**(2d) 3-[3-(1-Piperidinylmethyl)-4-hydroxybenzylidene]-5-(3,4-dimethoxybenzylidene)-4-piperidone dihydrochloride**

Yield: 30%. *^1^H NMR (DMSO-d_6_)*: δ 2.45 (t, 2H, -CH_2_-N-CH_2_), 3.19 (t, 2H, -CH_2_-N-CH_2_), 3.74 (s, 2H, Ar-CH_2_-N), 3.92 (s, 3H, -OCH_3_), 3.95 (s, 3H, -OCH_3_), 4.2 (d, 4H, 2× NCH_2_), 6,83-6.96 (m, 4H, Ar-H), 7.23-7.73 (s, 2H, 2× =CH), 7.33 (s, 2H, Ar-H). *^13^C NMR (DMSO-d_6_)*: 22.64, 40.55, 44.52, 52.13, 56.14, 112.23, 114.84, 124.71, 126.15, 126.92, 139.75, 149.15, 159.38, 182.44. *HRMS (ESI): (m/z)*: 448.2351 (M^+^), Calc: 448.2362 for C_27_H_32_N_2_O_4_, 449.2395 (M + 1), 450.2431 (M + 2)

**(2e) 3-[3-(4-Morpholinomethyl)-4-hydroxybenzylidene]-5-(3,4-dimethoxybenzylidene)-4-piperidone dihydrochloride**

Yield: 30%. ^1^H NMR (DMSO-d_6_): δ 2.55 (t, 4H, -CH_2_-N-CH_2_), 3.30 (t, 4H, -CH**_2_**-O-CH_2_), 3.64 (s, 2H, Ar-CH_2_-N), 3.75 (4H, 2× NCH_2_), 3.85–4.10 (m, 6H, 2× OCH_3_), 6.75–7.25 (m, 4H, Ar-H), 7.40 (s, 2H, Ar-H), 7.45 (s, 2H, 2× =CH). *^13^C NMR (DMSO-d_6_)*: 44.54, 56.02, 56.16, 63.49, 79.68, 112.41, 114.83, 120.04, 122.62, 127.99, 128.07, 139.87, 148.87, 149.14, 182.44. *HRMS (ESI): (m/z)*: 450.2169 (M^+^), Calc: 450.2154 for C_26_H_30_N_2_O_5_, 451.2201 (M + 1), 452.2265 (M + 2)

**(2f) 3-[3-(4-Methyl-1-piperazinylmethyl)-4-hydroxybenzylidene]-5-(3,4-dimethoxybenzylidene)-4-piperidone trihydrochloride**

Yield: 50%. *^1^H NMR (DMSO-d_6_*): δ 2.38 (s, 3H, N-CH**_3_**), 2.40–2.52, (8H, 2× -CH**_2_**-N-CH**_2_**), 3.75 (m, 4H, 2× NCH_2_), 3.78 (s, 2H, Ar-CH_2_-N), 3.85–3.90 (m, 6H, 2× OCH_3_), 6.84–6.90 (m, 4H, Ar-H), 7.02–7.08 (s, 2H, Ar-H), 7.75 (s, 2H, 2× =CH). *^13^C NMR (DMSO-d_6_)*: 39.45, 48.11, 55.02, 56.00, 58.64, 112.04, 114.43, 116.20, 123.21, 124.35, 128.23, 134.51, 148.94, 150.10, 158.59, 182.45. *HRMS (ESI): (m/z)*: 463.2465 (M^+^), Calc: For C_27_H_33_N_3_O_4_, 464.2491 (M + 1), 465.2515 (M + 2)

**(3a) 3-[4-Methyl-1-piperazinylmethyl-4-hydroxybenzylidene]-5-benzylidene-4-piperidone trihydrochloride**

Yield: 40%. *^1^H NMR (DMSO-d_6_*): δ 2.38 (s, 3H, N-CH_3_), 2.69 (br. s, 8H, 2× -CH_2_-N-CH_2_), 3.77 (s, 2H, Ar-CH_2_-N), 4.18 (s, 4H, 2× NCH_2_), 6.90–7.10 (m, 2H, Ar-H), 7.39 (s, 4H, Ar-H), 7.45 (m, 2H, Ar-H), 7.75–7.82 (s, 2H, 2× =CH). *^13^C NMR (DMSO-d_6_)*: 48.27, 61.13, 77.19, 99.08, 116.06, 128.31, 129.07, 130.60, 133.13, 135.65, 136.72, 141.92, 180.91. *HRMS (ESI): (m/z):* 403.2246 (M^+^), Calc: For C_25_H_29_N_3_O_2_, 404.2258 (M + 1), 406.2324 (M + 2)

**(3b) 3-[4-Methyl-1-piperazinylmethyl-4-hydroxybenzylidene]-5-(4-fluorobenzylidene)-4-piperidone trihydrochloride**

Yield: 45%. *^1^H NMR (DMSO-d_6_*): δ 2.39 (s, 3H, N-CH_3_), 2.72 (br. s, 8H, 2× -CH_2_-N-CH_2_), 3.72 (s, 2H, Ar-CH_2_-N), 4.20 (br. s, 4H, 2× NCH_2_), 6.78–7.16 (m, 4H, Ar-H), 7.33–7.42 (s, 3H, Ar-H), 7.70–7.86 (s, 2H, 2× =CH). *^13^C NMR (CDCl_3_)*: 47.97, 76.58, 115.15, 116.52, 131.82, 132.44, 134.27, 135.04, 136.72, 142.00, 151.26, 154.16, 172.92, 196.10. *HRMS (ESI): (m/z)*: 421.2155 (M^+^), Calc: For C_25_H_28_FN_3_O_2_, 422.2227 (M + 1), 423.2297 (M + 2)

**(3c) 3-[4-Methyl-1-piperazinylmethyl-4-hydroxybenzylidene]-5-(4-methoxybenzylidene)-4-piperidone trihydrochloride**

Yield: 50%. *^1^H NMR (DMSO-d_6_*): δ 2.38 (s, 3H, N-CH_3_) 2.68, (br. s, 8H, 2× -CH_2_-N-CH_2_), 3.73 (s, 2H, Ar-CH_2_-N), 3.84 (s, 3H, -OCH_3_), 4.17 (s, 4H, 2× NCH_2_), 6.75–7.00 (m, 4H, Ar-H), 7.08–7.32 (m, 3H, Ar-H), 7.70 (s, 1H, Ar-H), 7.72 (s, 1H, Ar-H). *HRMS (ESI): (m/z):* 433.2350 (M^+^), Calc: For C_26_H_31_N_3_O_3_, 434.2401 (M + 1), 435.2449 (M + 2)

**(3d) 3-[4-Methyl-1-piperazinylmethyl-4-hydroxybenzylidene]-5-(4-methylbenzylidene)-4-piperidone trihydrochloride**

Yield: 50%. *^1^H NMR (DMSO-d_6_*): δ 2.05 (s, 3H, N-CH_3_), 2.08 (t, 8H, 2× -CH_2_-N-CH_2_), 2.48 (s, 3H, Ar-CH_3_), 3.77 (s, 2H, -NCH_2_), 3.79 (s, H, -NCH_2_), 4.18 (s, H, Ar-CH_2_-N), 6.75-6.98 (m, 4H, Ar-H), 7.09–7.19 (m, 3H, Ar-H), 7.63–7.81 (m, 2H, 2× =CH). *^13^C NMR (CDCl_3_)*: 43.53, 45.82, 48.12, 60.67, 61.43, 77.35, 116.68, 122.18, 125.55, 129.22, 130.45, 131.52, 131.67, 135.65, 136.26, 190.89. *HRMS (ESI): (m/z):* 417.2396 (M^+^), Calc: For C_26_H_31_N_3_O_2_, 418.2419 (M + 1), 419.2472 (M + 2)

**(3e) 3-[4-Methyl-1-piperazinylmethyl-4-hydroxybenzylidene]-5-(4-nitrobenzylidene)-4-piperidone trihydrochloride**

Yield: 50%. *^1^H NMR (DMSO-d_6_*): δ 2.19 (s, 3H, N-CH_3_), 2.32–2.61 (m, 8H, 2× -CH_2_-N-CH_2_), 3.79 (s, 2H, Ar-CH_2_-N), 4.18 (s, H, -NCH_2_), 4.19 (s, H, -NCH_2_), 6.78–7.16 (m, 3H, Ar-H), 7.49–7.61 (m, 2H, Ar), 7.75–7.83 (m, 2H, Ar-H), 8.22–8.33 (m, 2H, 2× =CH). *^13^C NMR (CDCl_3_)*: 62.8, 77.35, 116.68, 123.71, 130.91, 131.98, 132.28, 137.49, 141.77, 190.74. *HRMS (ESI): (m/z):* 449.2191 (M^+^), Calc: For C_25_H_28_N_4_O_4_, 450.2255 (M + 1), 451.2318 (M + 2)

### 4.2. Bioevaluations

#### 4.2.1. Cytotoxicity Assays

A literature procedure was followed for the evaluation of **1**, **2a**–**f**, and **3a**–**e** against human colon cancer cell lines (HCT116, HT29) and human non-malignant colon cells (CRL1790) [25]. The culture medium was supplemented with 10% heat-inactivated fetal bovine serum (FBS) and 1% penicillin–streptomycin antibiotics. The time of the bioassays was 48 h. The determinations were undertaken in triplicate on three different occasions.

The method for evaluating **2a**–**f** and **3a**–**e** against human oral squamous cell carcinoma (OSCC) cell lines (Ca9-22, HSC-2, HSC-3, HSC-4) and human non-malignant oral cells [human gingival fibroblast (HGF), human periodontal ligament fibroblast (HPLF), and human pulp cell (HPC)] have been reported previously [24], except the length of time of this bioassay was extended from 24 h to 48 h. In brief, different quantities of the compounds were incubated with the cells in Dulbecco’s modified eagle medium (DMEM), which was supplemented with 10% FBS and antibiotics. The cell viability was determined by the 3-(4,5-dimethylthiazol-2-yl)-2,5-diphenyl-2H-tetrazolium bromide (MTT) method as summarized previously [24] (Figure 4). Apoptotic cells that showed a shrunken morphology were visualized under light microscopy (EVOSfl; ThermoFisher Scientific, Waltham, MA, USA) (Figure 5A).

The method used for generating the data of human acute lymphocytic leukemia (CEM) and human acute promyelocytic leukemia (HL-60) cells in Table 4 has been described previously [26]. In brief, the cells were cultured in media supplemented with 10% FBS and antibiotics. Cell viability was measured using the differential DNA staining (DNS) assay after 24 h incubation [27,28]. The CC_50_ values of the compounds listed in Table 4 were obtained by linear interpolation equations [28,29].

#### 4.2.2. Bioassays

Apoptosis induction in Ca9-22 cells was evaluated by cell cycle analysis and Western blot analysis as described previously [25,28] (Figure 5). Briefly, for the cell cycle analysis, cells were treated for 24 h with the test samples. Cells were then fixed, digested with RNase A, stained with propidium iodide, filtered through Falcon^®^ cell trainers (Corning Inc., Corning, NY, USA), and then subjected to cell sorting. For the Western blot, cell lysates (equivalent to 5 µg protein) were applied to SDS-polyacrylamide gel electrophoresis. A literature method was used to determine whether representative compounds led to increases in the production of ROS [29]. In brief, CEM cells were treated with the compounds overnight, then with 6-carboxy-2′,7′-dichlorodihydrofluorescein diacetate (carboxy-H2DCFDA) and the samples were processed using flow cytometry. The data generated are presented in Figure 6A.

In order to discover if representative compounds induce apoptosis, the annexin V-FITC assay was used [29]. In brief, CEM cells were treated with various compounds for 24 h. The cells were collected and stained with propidium iodide and annexin V-FITC. The results were obtained using flow cytometry and are presented in Figure 6B.

The effect on the mitochondrial membrane potential in CEM cells was undertaken using a published method [29]. In brief, the cells were incubated with different compounds for 7 h. The cells were then stained with the JC-1 dye, and the samples were examined by flow cytometry. The results are portrayed in Figure 6C.

### 4.3. ADME Determination

The data generated in Table 6 were obtained using SwissADME (Swiss Institute of Bioinformatics, Lausanne, Switzerland) [30].

### 4.4. Statistical Treatment

Statistical analyses were performed using the SPSS 23.0 software (IBM, Armonk, NY, USA). Experimental data are presented as the mean ± standard deviations (SD) of triplicate determinations. The significance of values was examined by one-way analysis of variance (ANOVA) and appropriate Bonferroni’s post-test. A value of * *p* < 0.05 was considered to indicate statistically significant differences.

## 5. Conclusions

1. This study revealed that the compounds in series **2** and **3** are novel potent cytotoxins. In several instances, their potencies exceed that of established anticancer agents. A further positive feature is their greater toxicity to tumors than non-malignant cells in a number of bioassays.
2. The modes of action whereby representative compounds exert their bioactivity was determined in some cases.
3. The compounds are toxic to different clusters of cancers. In the future, this section of the project needs to be expanded in order to evaluate the efficacy of **2a**–**f** and **3a**–**e** on such important tumors as breast, lung, and prostate cancers.
4. Some support was gathered for the hypothesis that SI values are influenced by differences in the atomic charges on the olefinic carbon atoms. In other words, sufficient encouragement has been found to continue evaluating this theory with other clusters of compounds and different biological targets.

## Figures and Tables

**Table 1:** Evaluation of **1**, **2a**–**f**, and **3a**–**e** against human HCT116 and HT29 colon cancer cells and human CRL1790 colon cells.

Compound	HCT116		HT29		Average		CRL1790	PSE ^b^
	IC_50_ (µM)	SI ^a^	IC_50_ (µM)	SI ^a^	IC_50_	SI	IC_50_ (µM)	
**1**	3.27 ± 0.10	1.07	0.61 ± 0.06	5.74	1.94	3.41	3.50 ± 0.28	176
**2a**	1.73 ± 0.48	10.1	1.80 ± 1.24	9.72	1.77	9.91	17.5 ± 0.58	560
**2b**	0.20 ± 0.03	76.0	0.29 ± 0.08	52.4	0.25	64.2	15.2 ± 0.15	25,680
**2c**	3.44 ± 0.42	7.67	1.57 ± 0.54	16.8	2.51	12.2	26.4 ± 0.28	486
**2d**	0.45 ± 0.15	52.2	0.54 ± 0.17	43.5	0.50	47.9	23.5 ± 0.26	9580
**2e**	3.37 ± 0.68	8.13	3.75 ± 0.78	7.31	3.56	7.72	27.4 ± 0.42	217
**2f**	0.68 ± 0.31	18.7	0.76 ± 0.30	16.7	0.72	17.7	12.7 ± 0.31	2458
**3a**	0.33 ± 0.11	65.8	0.40 ± 0.03	54.3	0.37	60.1	21.7 ± 0.25	16,243
**3b**	0.28 ± 0.16	45.0	0.26 ± 0.08	48.5	0.27	46.8	12.6 ± 0.24	17,333
**3c**	1.04 ± 0.48	12.7	0.76 ± 0.26	17.4	0.90	15.1	13.2 ± 0.36	1678
**3d**	3.91 ± 1.69	5.78	0.71 ± 0.29	31.8	2.31	18.8	22.6 ± 0.59	814
**3e**	0.34 ± 0.09	46.5	0.32 ± 0.07	49.4	0.33	48.0	15.8 ± 0.27	14,546
5-FU	3.54 ± 0.72	4.97	14.4 ± 0.65	1.22	8.97	3.10	17.6 ± 0.82	34.6

^a^ The letters SI refer to the selectivity index, which is the ratio of the IC_50_ values of the compound against CRL1790 cells and the IC_50_ figures against either HCT116 or HT29 cells. ^b^ The letters PSE stand for the potency–selectivity expression, which is the reciprocal of the average potency towards neoplastic cells multiplied by the average SI values times 100.

**Table 2:** Evaluation of **2a**–**f** and **3a**–**e** against human Ca9-22, HSC-2, HSC-3, and HSC-4 squamous cell carcinoma cell lines.

Compound	Ca9-22		HSC-2		HSC-3		HSC-4		Average	
	CC_50_^a^	SI ^b^	CC_50_^a^	SI ^b^	CC_50_^a^	SI ^b^	CC_50_^a^	SI ^b^	CC_50_^a^	SI ^b^
**2a**	0.68	11.4	1.20	6.44	1.06	7.29	1.39	5.56	1.08	7.67
**2b**	0.65	10.8	1.01	6.96	0.76	9.25	1.16	6.06	0.90	8.27
**2c**	8.20	6.54	14.0	3.83	11.7	4.58	14.1	3.80	12.0	4.69
**2d**	0.42	<8.98	0.61	<6.18	0.39	<9.67	0.75	5.03	0.54	7.47
**2e**	41.8	>4.79	88.5	>2.26	73.2	>2.73	>3.7	2.71	69.3	3.12
**2f**	1.94	8.81	2.80	6.11	2.47	6.92	2.73	6.26	2.49	7.03
**3a**	0.43	13.6	0.67	8.70	0.49	11.9	0.71	8.21	0.58	10.6
**3b**	0.55	18.4	0.91	11.1	0.95	10.6	1.14	8.86	0.89	12.2
**3c**	0.66	8.33	1.10	5.00	1.05	5.24	1.18	4.66	1.00	5.81
**3d**	1.30	7.85	2.20	4.64	1.91	5.34	2.29	4.45	1.93	5.57
**3e**	0.33	17.3	0.58	9.83	0.47	12.1	0.60	9.50	0.50	12.2
5-FU ^c^	141	>7.09	359	>2.79	450	>2.22	7.8	>128	>240	>35.0
MPN ^c^	42.7	>4.68	17.0	>11.8	20.8	>9.62	10.4	>19.2	22.7	>11.3
CDDP ^c^	212	>4.72	204	>4.90	46.9	>21.3	60.4	>16.6	131	>11.1
DXR ^c^	0.33	>30.3	0.20	>50.0	0.24	>41.7	0.14	>71.4	0.23	>48.4

^a^ The CC_50_ values are in µM and are the concentrations of the compounds required to kill 50% of the cells. ^b^ The letters SI refer to the selectivity index figures, which are quotients of the average CC_50_ values towards HGF, HPLF, and HPC non-malignant cells (Table 3) and the CC_50_ values against a specific neoplastic cell line. ^c^ The designations 5-FU, MPN, CDDP, and DXR refer to 5-flurouracil, melphalan, cisplatin, and doxorubicin, respectively.

**Table 3:** Evaluation of **2a**–**f** and **3a**–**e** against three non-malignant oral cell lines.

Compound	CC_50_ (µM) ^a^				PSE ^b^
	HGF	HPLF	HPC	Average	
**2a**	15.9	5.0	2.3	7.73	710
**2b**	13.3	5.5	2.3	7.03	951
**2c**	68.6	48.9	43.3	53.6	39.1
**2d**	7.0	2.7	<1.6	<3.77	1383
**2e**	>200	>200	>200	>200	>4.50
**2f**	33.1	12.0	6.1	17.1	282
**3a**	10.2	4.7	2.6	5.83	1828
**3b**	18.5	7.4	4.3	10.1	1371
**3c**	8.8	4.9	2.8	5.50	593
**3d**	14.6	10.1	6.0	10.2	289
**3e**	12.5	2.7	1.9	5.70	2440
5-FU ^c^	>1000	>1000	>1000	>1000	>14.6
MPN ^c^	>200	>200	>200	>200	>49.8
CDDP ^c^	>1000	>1000	>1000	>1000	>8.47
DXR ^c^	>10	>10	>10	>10	>21,044

^a^ The CC_50_ values are the concentrations of the compounds required to kill 50% of the cells. ^b^ The potency–selectivity expression (PSE) is the product of the reciprocal of the average CC_50_ values towards Ca9-22, HSC-2, HSC-3, and HSC-4 cells and the average SI values times 100. ^c^ The abbreviations 5-FU, MPN, CDDP, and DXR refer to 5-fluorouracil, melphalan, cisplatin, and doxorubicin, respectively.

**Table 4:** Evaluation of **2a**–**c**, **f**, **3a**, **b**, and **e** against CEM and HL-60 cells.

Compound	CC_50_ (µM) CEM Cells		CC_50_ (µM) HL-60 Cells	
	24 h	48 h	24 h	48 h
**2a**	1.64 ± 0.68	0.82 ± 0.02	1.99 ± 0.92	2.21 ± 0.37
**2b**	0.63 ± 0.08	0.82 ± 0.03	7.75 ± 0.26	4.82 ± 0.91
**2c**	>10	7.92 ± 0.98	>10	>10
**2f**	8.91 ± 1.32	2.89 ± 0.20	3.75 ± 1.07	2.73 ± 0.57
**3a**	3.06 ± 0.08	3.32 ± 0.30	8.27 ± 0.40	1.67 ± 0.54
**3b**	3.15 ± 1.29	0.40 ± 0.03	5.90 ± 0.76	2.65 ± 0.43
**3e**	0.76 ± 0.15	0.83 ± 0.03	4.11 ± 24.5	6.63 ± 0.68

**Table 5:** Plots between the average SI values in Table 1 and Table 2 and the differences between the atomic charges on C^A^ and C^B^.

Average SI Data	Charges	Plots ^a^	Correlations (*p* < 0.05) and Trends to a Correlation (*p* < 0.1)
**1, 2a–f, 3a–e**	Mulliken	l	*p* < 0.05
	Mulliken	sl	*p* < 0.10
	Natural	log	*p* < 0.10
**2a–f, 3a–e**	Electrostatic	l	*p* < 0.05
	Natural	l	*p* < 0.05
	Electrostatic	sl	*p* < 0.05
	Natural	sl	*p* < 0.10

^a^ The plots made are linear (l), semilogarithmic (sl), or logarithmic (log).

**Table 6:** Evaluation of **2b, 2d** and **3a**–**c** for some drug-like properties.

Compound	MW (g/mol) ^a^	log P ^a^	HBA ^a^	HBD ^a^	RB ^a^	TPSA (Å^2^) ^a^
**2b**	436.54	3.14	6	2	8	71.03
**2d**	448.55	2.90	6	2	6	71.03
**3a**	403.52	1.27	5	2	4	55.81
**3b**	421.51	1.83	6	2	4	55.81
**3c**	433.54	1.28	6	2	5	65.04
Ideal Compound	≯500	≯5	≯10	≯5	<10	<140 Å^2^

^a^ The abbreviations in the headings of each column refer to molecular weight (MW), the logarithm of the partition coefficient (log P), hydrogen-bond-acceptor atoms (HBA), hydrogen-bond-donor atoms (HBD), rotatable bonds (RB), and total polar surface area (TPSA).

## Data Availability

Not applicable.

## Supplementary Materials

The following supporting information can be downloaded at: https://www.mdpi.com/article/10.3390/molecules27196718/s1, Table S1: The atomic charges on the olefinic carbon atoms of **1**, **2a**–**f**, and **3a**–**e**. Figure S1: Dose–response curves of the toxicities of **1**, **2a**–**f** and **3a**–**e** against OSCC cell lines and non-malignant cells. Figure S2: Dose-dependent accumulation of the subG1 population by **2b** in Ca9-2 cells. Spectra S1: ^1^H NMR spectra of compounds **2a**–**d** and **3a**–**e**. Spectra S2: ^13^C NMR spectra of compounds **2a**–**d**, **3a**, **3b**, **3d**, and **3e**. Spectra S3: Mass spectra of compounds **2a**–**f** and **3a**–**e**.

## Sample Availability

The samples are un-available from the authors.
