# Supplementary material for: Novel Unsymmetric 3,5-Bis(benzylidene)-4-piperidones That Display Tumor-Selective Toxicity
Source: Molecules. 2022 Oct 9;27(19):6718. doi: 10.3390/molecules27196718 (PMC9572513; doi:10.3390/molecules27196718)
Supplement: Supplementary file 1 [file molecules-27-06718-s001.zip › molecules-1903800-supplementary.pdf]

SUPPLEMENTAL SECTION

Novel Unsymmetric 3,5-Bis(benzylidene)-4-piperidones That Display Tumor-Selective Toxicity

Aruna Chhikara [1], Praveen K. Roayapalley [2], Hiroshi Sakagami [3], Shigeru Amano [3], Keitaro Satoh [3], Yoshihiro Uesawa [4], Umashankar Das [2], Swagatika Das [2], Edgar A. Borrego [5], Cristina D. Guerena [5], Clare R. Hernandez [5], Renato J. Aguilera [5] and Jonathan R. Dimmock [2,*]

[1] Department of Chemistry, Dyal Singh College, University of Delhi, New Delhi 110003, India
[2] Drug Discovery and Development Research Cluster, College of Pharmacy and Nutrition, University of Saskatchewan, Saskatoon, SK S7N 5E5, Canada
[3] School of Dentistry, Meikai University, Sakado 350-0283, Japan
[4] Department of Medical Molecular Informatics, Meiji Pharmaceutical University, Tokyo 204-8588, Japan
[5] Department of Biological Sciences and Border Biomedical Research Center, The University of Texas at El Paso, El Paso, TX 79968-0519, USA

1. **Table S1**. Atomic charges on the olefinic carbon atoms of **1**, **2a**-**f**, **3a**-**e**.. S2
2. **Figure S1**. Concentration-dependent curves of **2a**-**f**, **3a**-**e** against human OSCC cell lines S3
3. **Figure S2**. Concentration-dependent accumulation of the subG1 population by **2b** in Ca9-22 cells.. S4
4. **Spectra S1**. ^{1}H NMR spectra of compounds **2a**-**d** and **3a**-**e**... S5
5. **Spectra S2**. ^{13}C NMR spectra of compounds **2a**-**d**, **3a**, **3b**, **3d**, and **3e**... S14
6. **Spectra S3**. Mass spectra of compounds **2a**-**f** and **3a**-**e**... S22

Table S1. Atomic charges on the olefinic carbon atoms of **1**, **2a**-**f**, **3a**-**e**

Compound	Electrostatic charges			Mulliken charges			Natural charges		
	νC^A	νC^B	$\Delta\nu$	νC^A	νC^B	$\Delta\nu$	νC^A	νC^B	$\Delta\nu$
**1**	-0.326	-0.331	0.005	-0.254	-0.254	0.000	-0.141	-0.142	0.001
**2a**	-0.272	-0.326	0.054	-0.246	-0.242	0.004	-0.144	-0.143	0.001
**2b**	-0.323	-0.322	0.001	-0.243	-0.255	0.012	-0.149	-0.139	0.002
**2c**	-0.318	-0.291	0.027	-0.249	-0.240	0.009	-0.149	-0.139	0.010
**2d**	-0.288	-0.309	0.021	-0.245	-0.243	0.002	-0.144	-0.141	0.003
**2e**	-0.283	-0.310	0.027	-0.245	-0.244	0.001	-0.146	-0.142	0.004
**2f**	-0.282	-0.312	0.030	-0.245	-0.243	0.002	-0.147	-0.141	0.006
**3a**	-0.232	-0.342	0.110	-0.237	-0.245	0.008	-0.137	-0.154	0.017
**3b**	-0.303	-0.395	0.092	-0.251	-0.258	0.007	-0.135	-0.152	0.017
**3c**	-0.221	-0.284	0.063	-0.239	-0.241	0.002	-0.142	-0.146	0.004
**3d**	-0.325	-0.357	0.032	-0.242	-0.244	0.002	-0.139	-0.151	0.012
**3e**	-0.311	-0.421	0.110	-0.251	-0.249	0.002	-0.128	-0.172	0.044

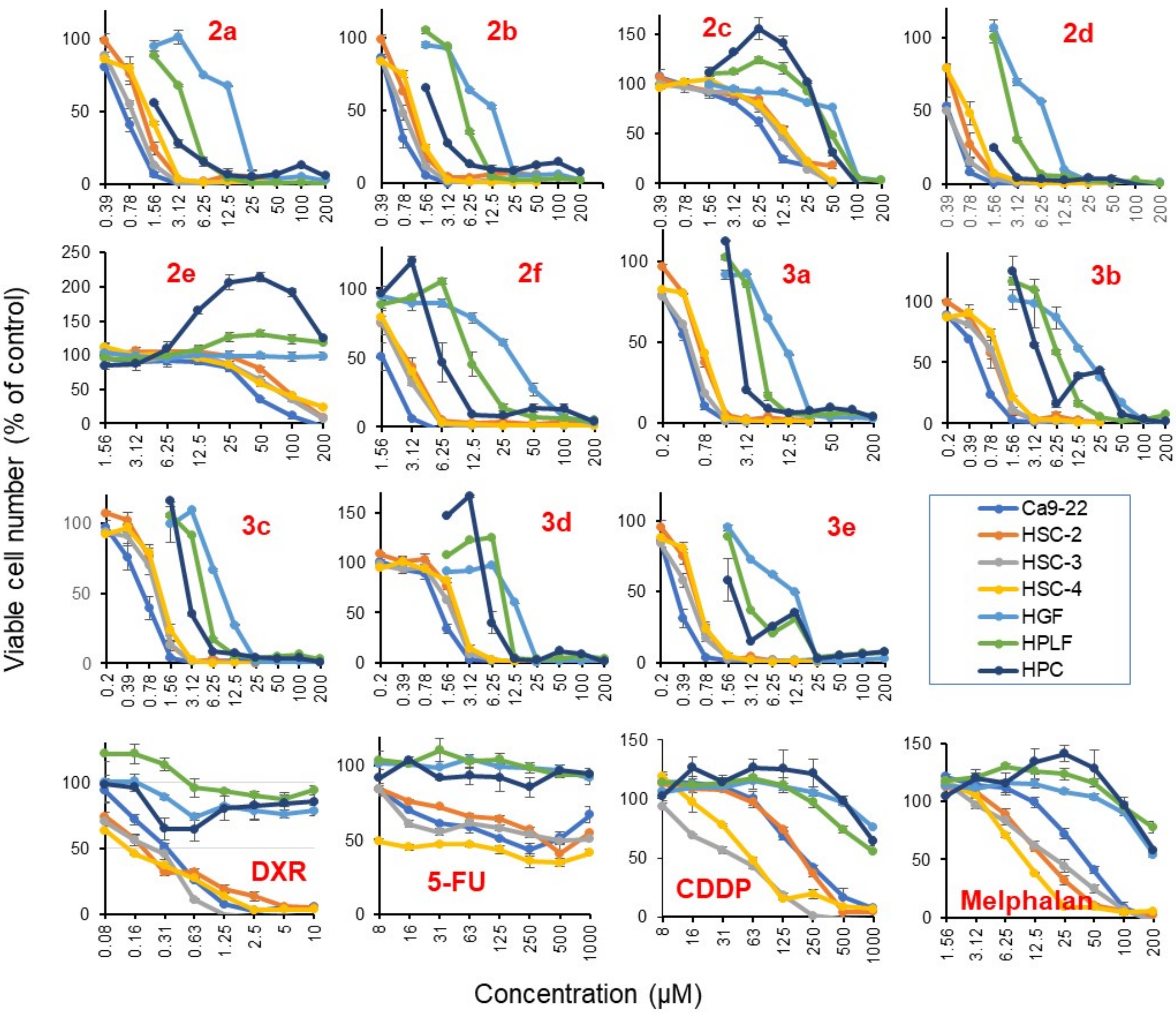

Figure S1. Concentration-dependent curves of **2a**-**f**, **3a**-**e** against human OSCC cell lines. Cells were incubated for 48h as controls or varying concentrations of the test compounds. Viable cell numbers, determined by the MTT method, are expressed as the percentage of the control. Each value is the mean ± S.D. of triplicate determinations.

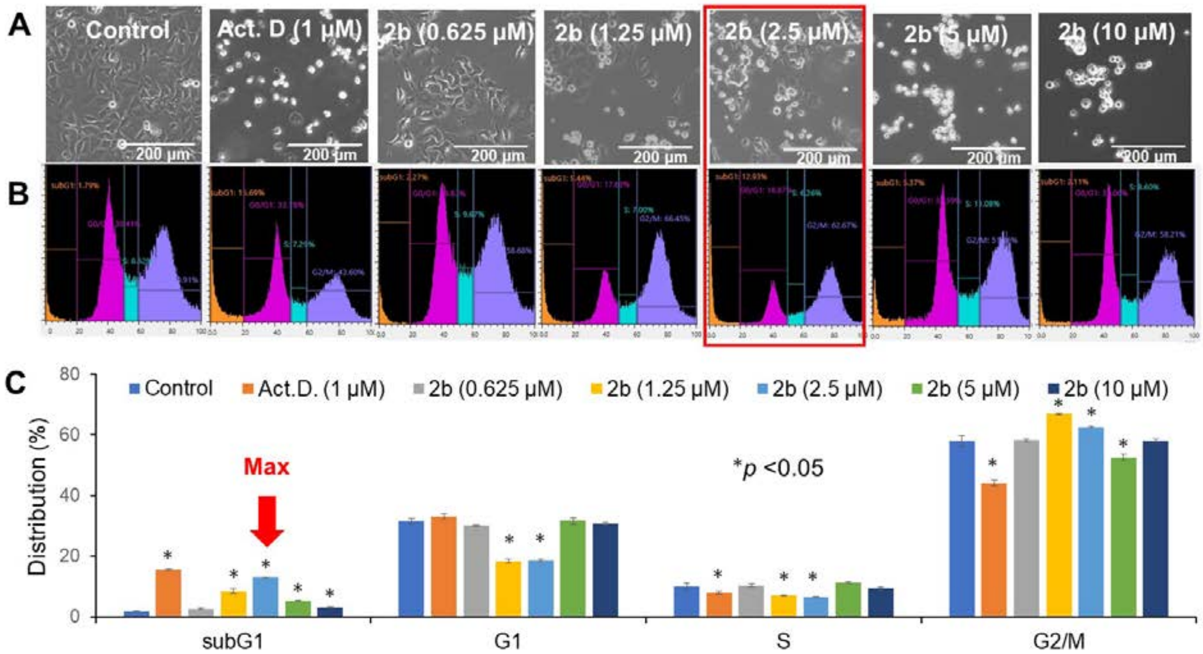

Figure S2. Concentration-dependent accumulation of the subG1 population by **2b** in Ca9-22 cells. Dose-dependent accumulation of the subG1 population by **2b** in Ca9-2 cells. Ca9-22 cells were incubated for 24 h with vehicle (0.1% DMSO), actinomycin D (1 μM), or **2b** (0.625, 1.25, 2.5, 5, 10 μM), and subjected to morphological observation [under light microscopy (EVOSfl; ThermoFisher Scientific, Waltham, MA, USA)] (**A**) and cell cycle analysis (**B**, **C**). Each value is represented as mean ± S.D. of triplicate determinations. *p<0.05 vs. control (Bonferroni's post-test) (**C**).

Spectra S1. ^{1}H NMR spectra of compounds **2a**-**d**, and **3a**-**e**.

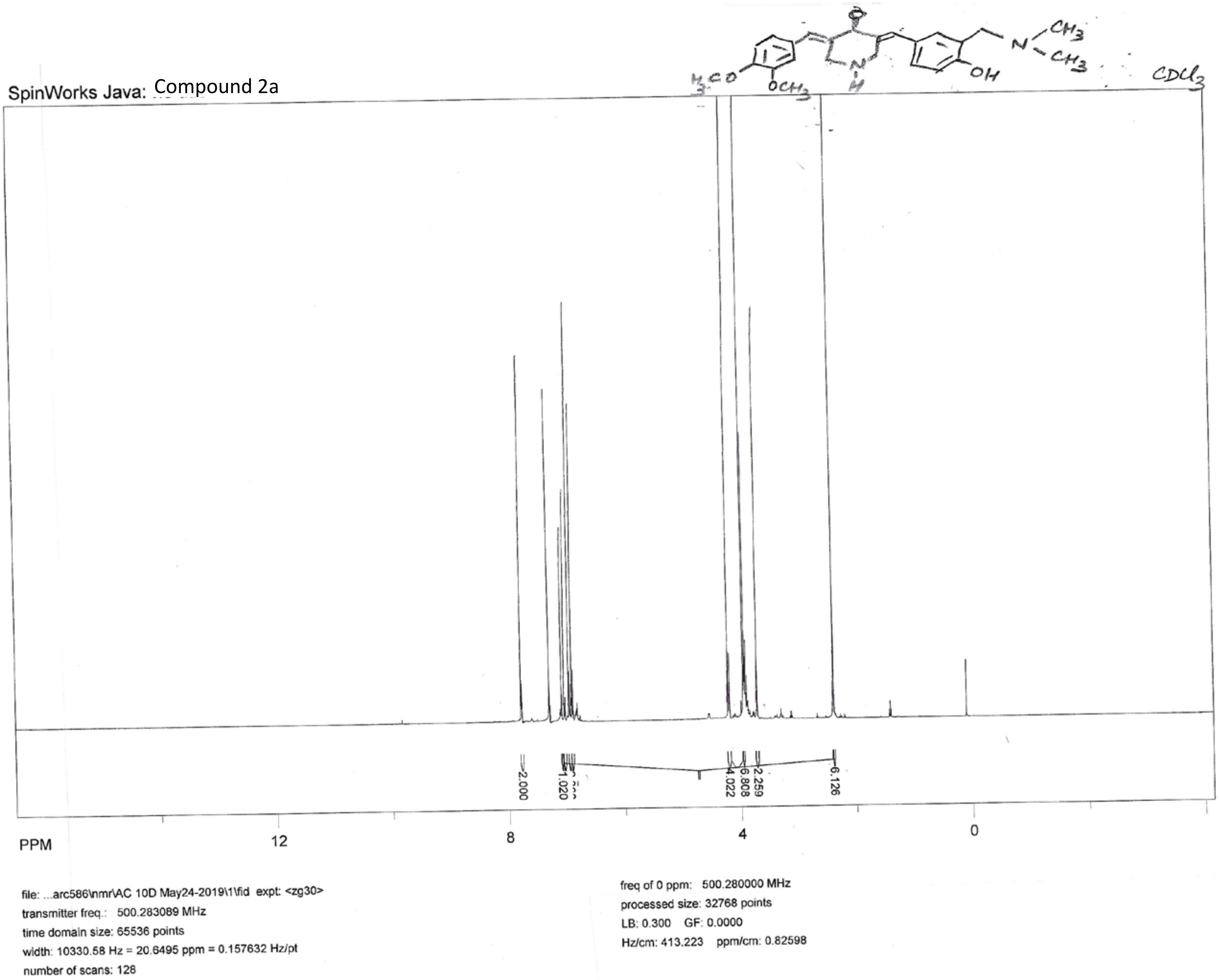

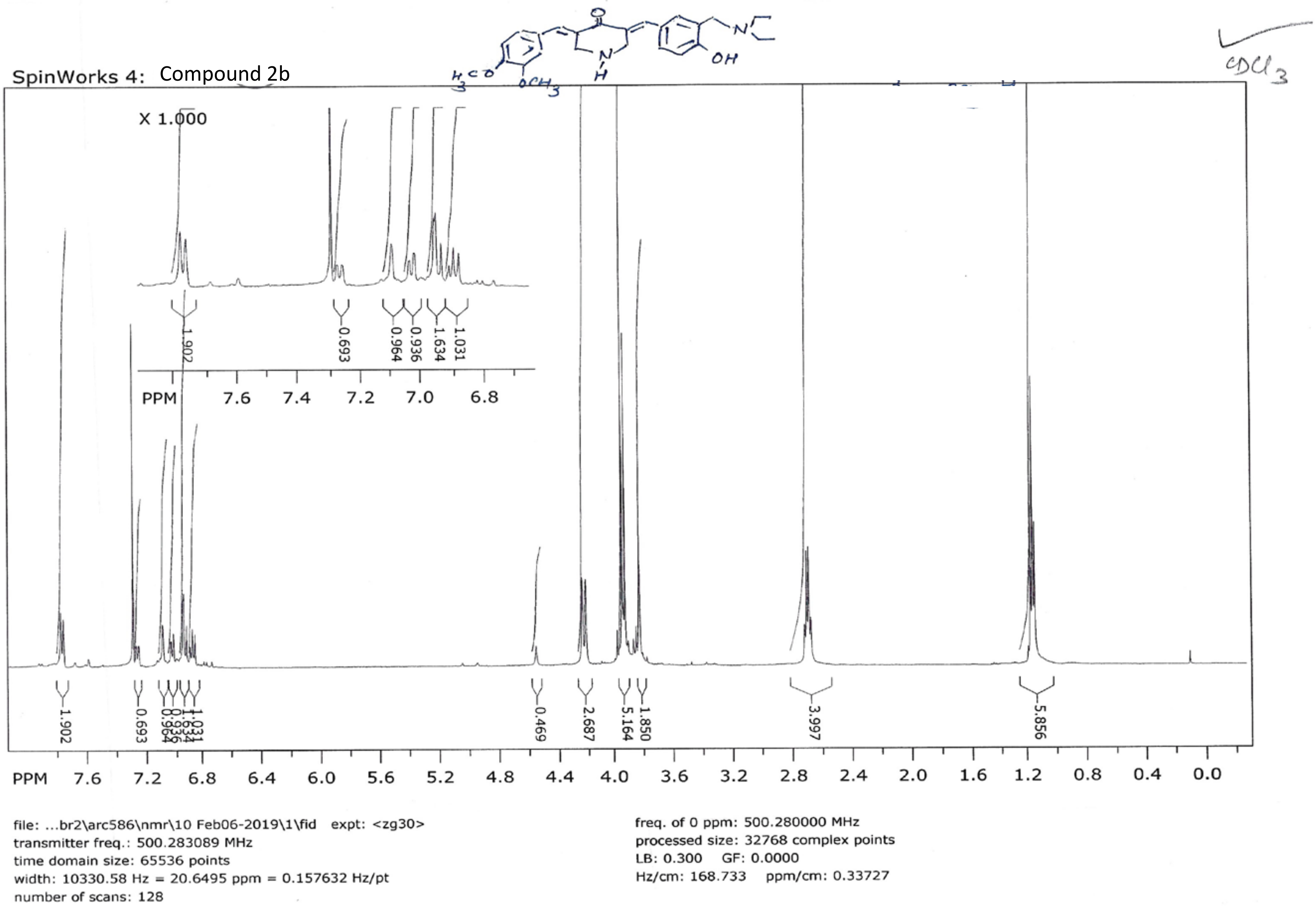
SpinWorks 4: Compound 2b
X 1.000
H_3CO
OCH_3
OH
CDCl3
1.902
0.693
0.964
0.936
1.634
1.031
0.469
2.687
5.164
1.850
3.997
5.856
PPM 7.6 7.2 6.8 6.4 6.0 5.6 5.2 4.8 4.4 4.0 3.6 3.2 2.8 2.4 2.0 1.6 1.2 0.8 0.4 0.0
file: ...br2\arc586\nmr\10 Feb06-2019\1\fid expt: <zg30>
transmitter freq.: 500.283089 MHz
time domain size: 65536 points
width: 10330.58 Hz = 20.6495 ppm = 0.157632 Hz/pt
number of scans: 128
freq. of 0 ppm: 500.280000 MHz
processed size: 32768 complex points
LB: 0.300 GF: 0.0000
Hz/cm: 168.733 ppm/cm: 0.33727

SpinWorks Java: [illegible] Compound 2c

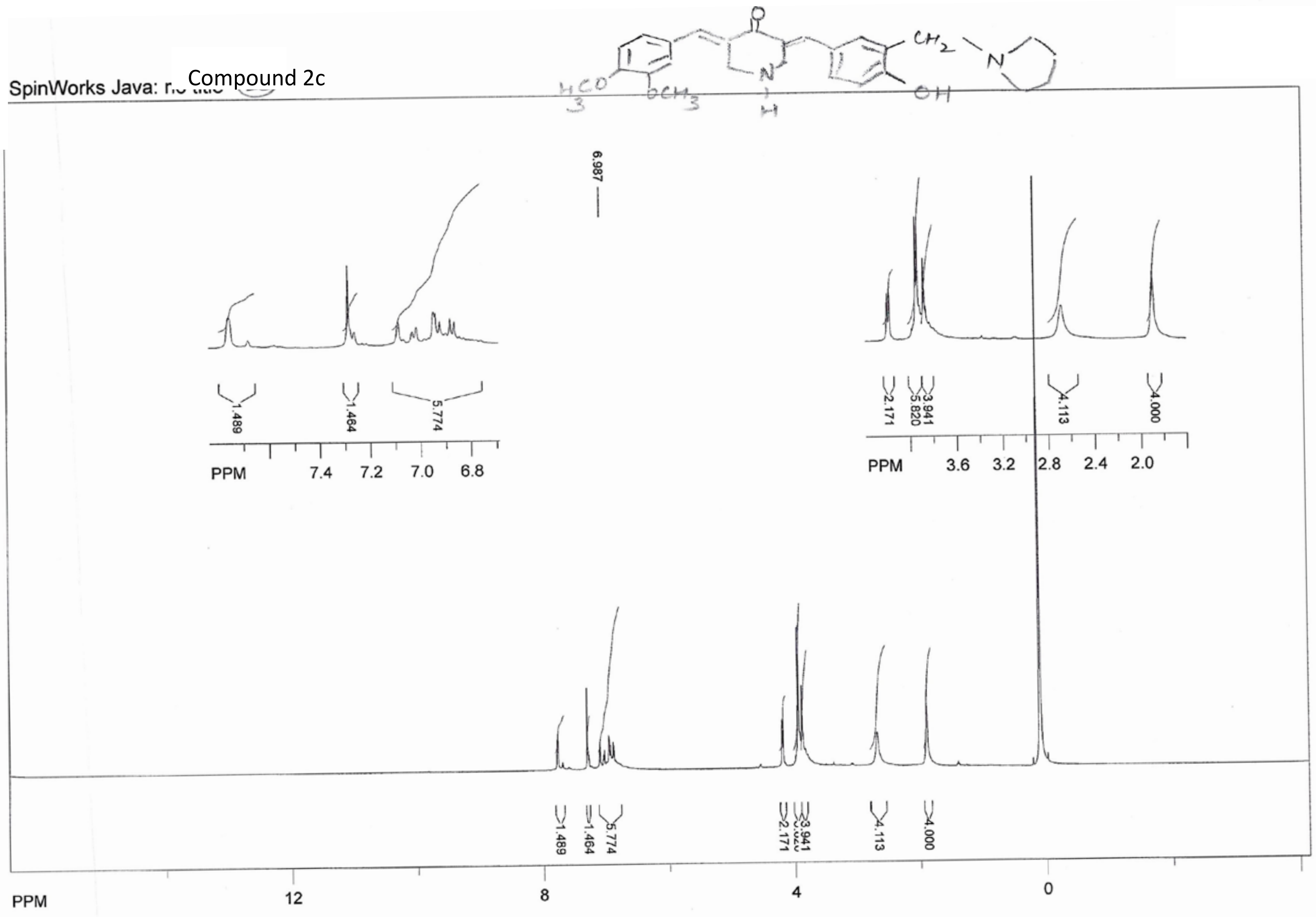

file: E:\FBR2\1\fid expt: <zg30> 10B $CDCl_3$
transmitter freq.: 500.283089 MHz
time domain size: 65536 points March14, 2019
width: 10330.58 Hz = 20.6495 ppm = 0.157632 Hz/pt
number of scans: 16

freq of 0 ppm: 500.280000 MHz
processed size: 32768 points
LB: 0.300 GF: 0.0000
Hz/cm: 413.223 ppm/cm: 0.82598

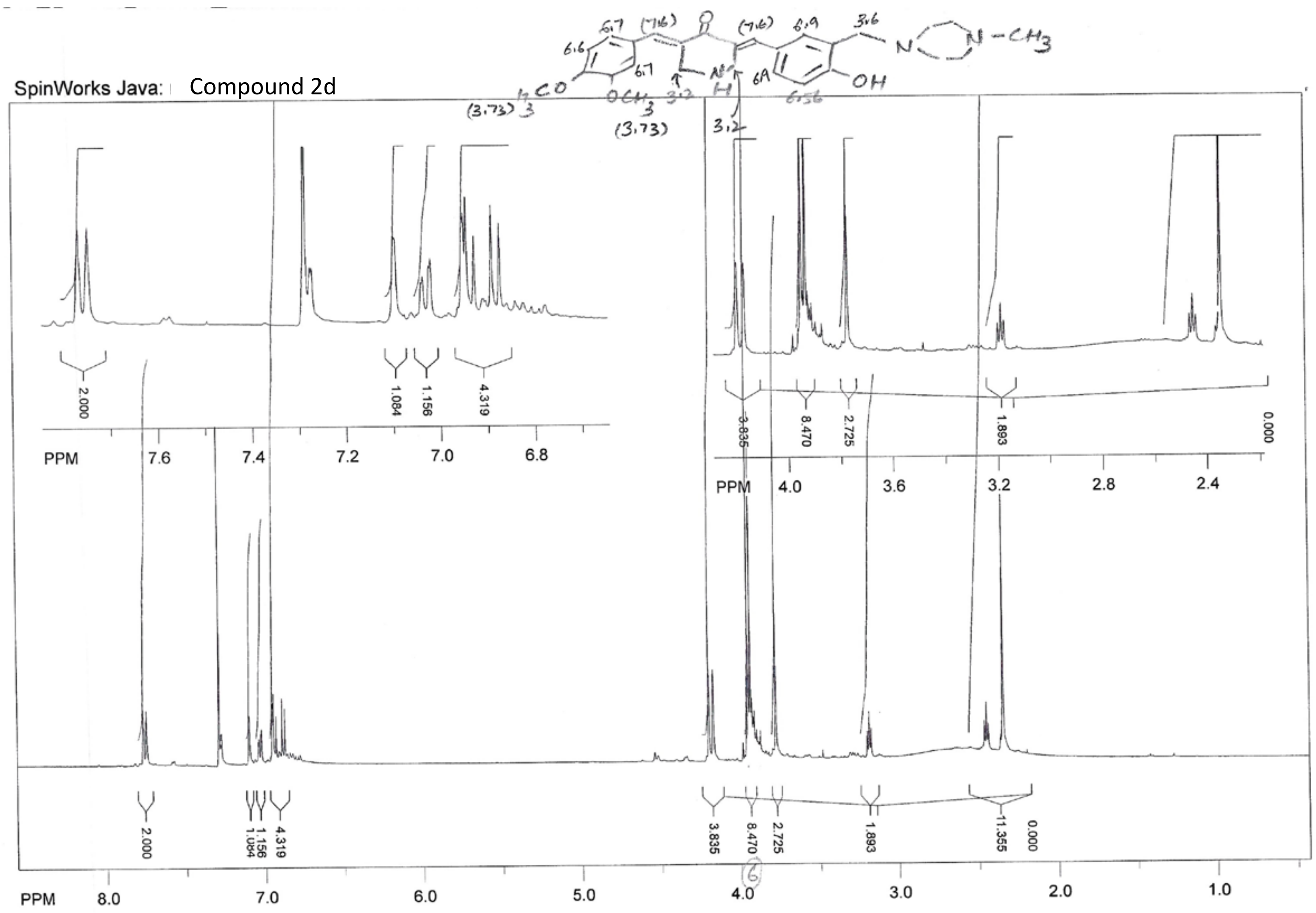

file: ...r2\arc586\nmr\10E Apr15-2019\1\fid expt: <zg30>
transmitter freq.: 500.283089 MHz
time domain size: 65536 points
width: 10330.58 Hz = 20.6495 ppm = 0.157632 Hz/pt
number of scans: 128

freq of 0 ppm: 500.280000 MHz
processed size: 32768 points
LB: 0.300 GF: 0.0000
Hz/cm: 162.924 ppm/cm: 0.32566

SpinWorks Compound 3a

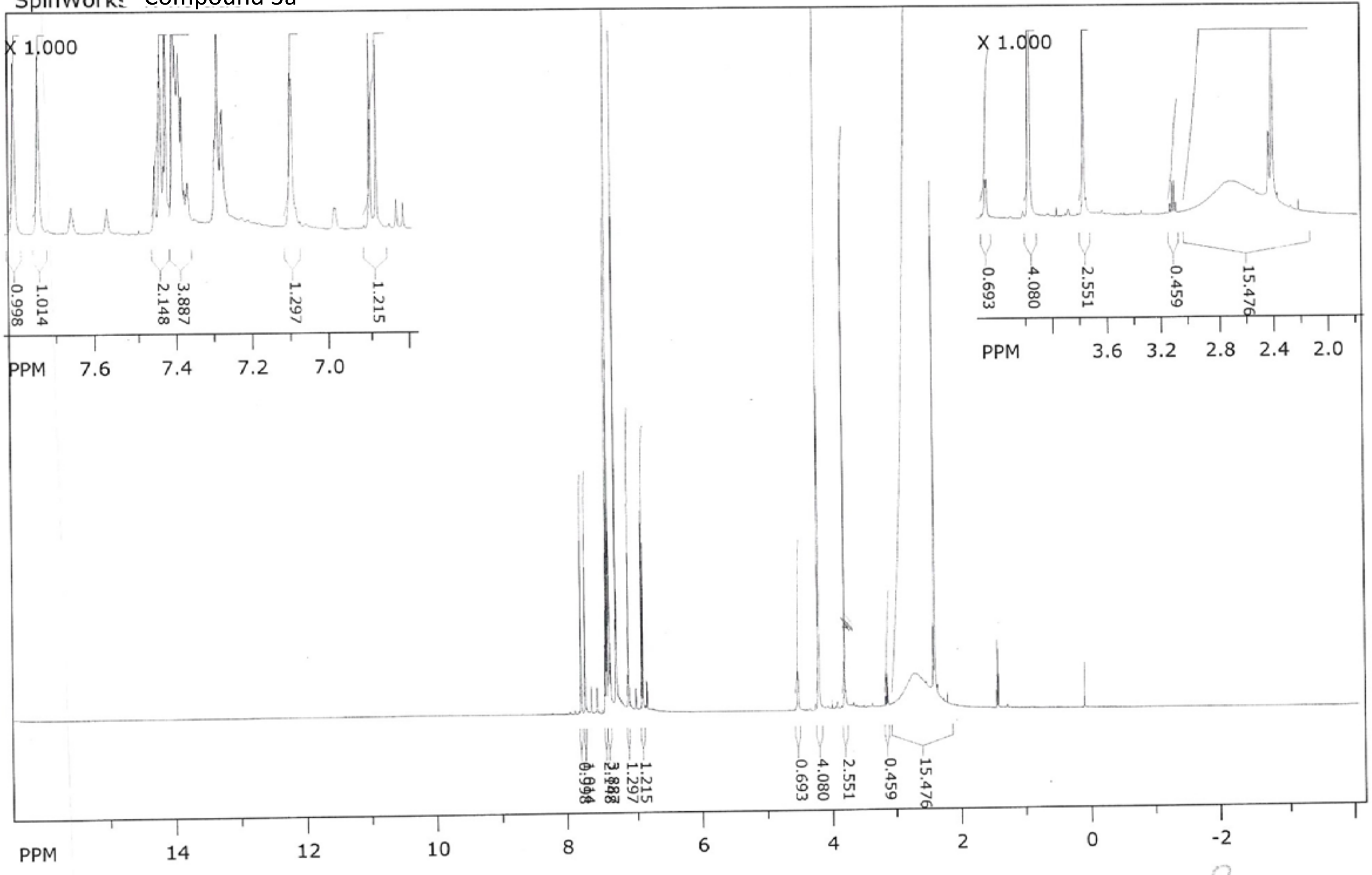

file: ...nzaldehyde deriv. Jan29-2020\1\fid expt: <zg30>
transmitter freq.: 500.283089 MHz
time domain size: 65536 points
width: 10330.58 Hz = 20.6495 ppm = 0.157632 Hz/pt
number of scans: 128

freq. of 0 ppm: 500.280000 MHz
processed size: 32768 complex points
LB: 0.300 GF: 0.0000
Hz/cm: 413.223 ppm/cm: 0.82598

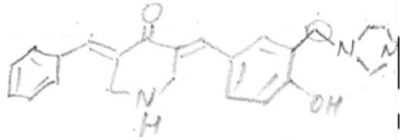

SpinWorks 4: Compound 3b

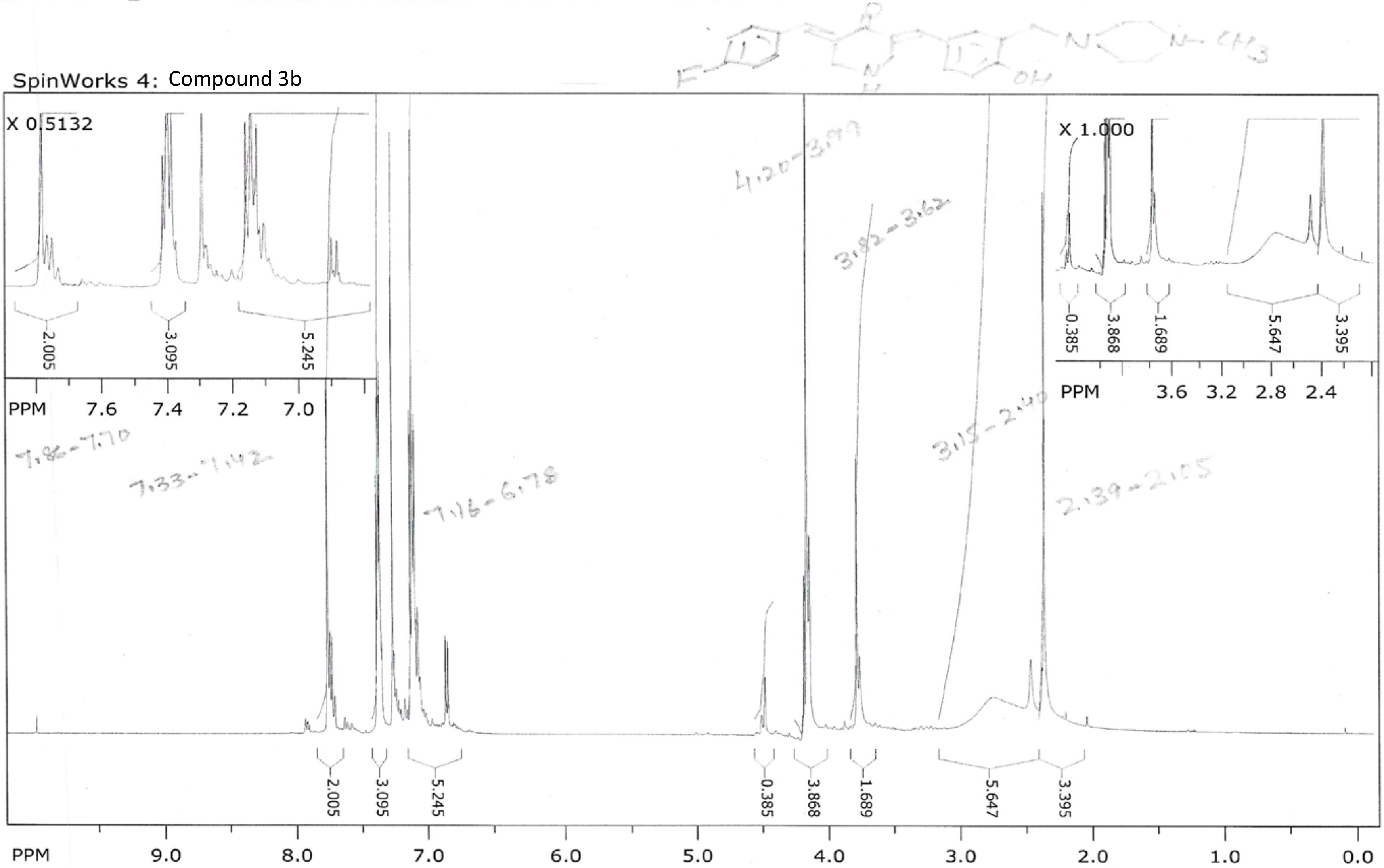

file: ...AC 2E para Fluoro Nov26-2019\1\fid expt: <zg30>
transmitter freq.: 500.283089 MHz
time domain size: 65536 points
width: 10330.58 Hz = 20.6495 ppm = 0.157632 Hz/pt
number of scans: 128

freq. of 0 ppm: 500.280000 MHz
processed size: 32768 complex points
LB: 0.300 GF: 0.0000
Hz/cm: 207.141 ppm/cm: 0.41405

SpinWorks 4: Compound 3c

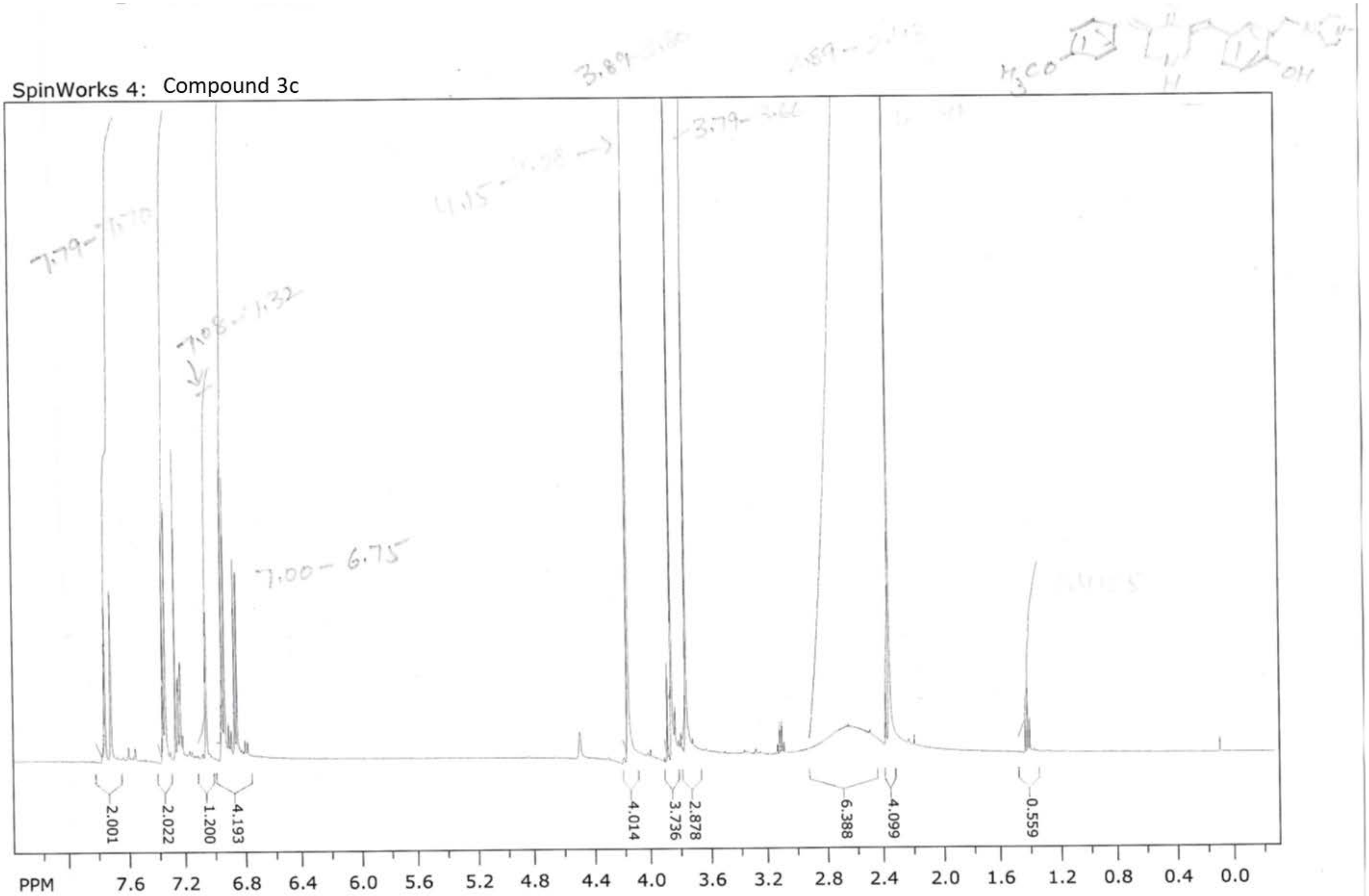

file: ...ta\u\fbr2\arc586\nmr\2700 AC\1\fid expt: <zg30>
transmitter freq.: 500.283089 MHz
time domain size: 65536 points
width: 10330.58 Hz = 20.6495 ppm = 0.157632 Hz/pt
number of scans: 128

freq. of 0 ppm: 500.280000 MHz
processed size: 32768 complex points
LB: 0.300 GF: 0.0000
Hz/cm: 174.031 ppm/cm: 0.34786

SpinWorks 4: Compound 3d

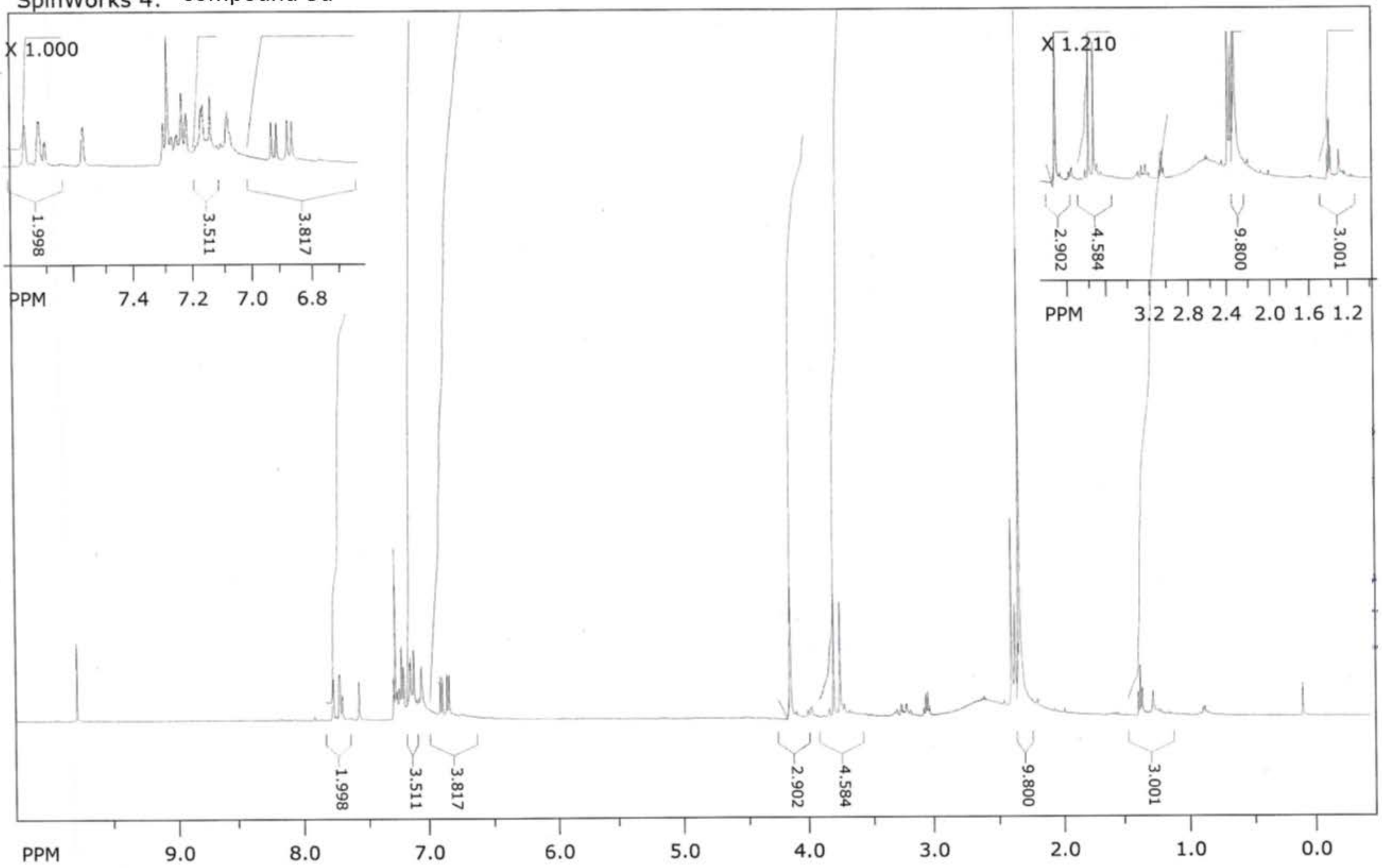

file: ...\u\fbr2\arc586\nmr\2701 AC 1\1\fid expt: <zg30>
transmitter freq.: 500.283089 MHz
time domain size: 65536 points
width: 10330.58 Hz = 20.6495 ppm = 0.157632 Hz/pt
number of scans: 128

freq. of 0 ppm: 500.280000 MHz
processed size: 32768 complex points
LB: 0.300 GF: 0.0000
Hz/cm: 215.088 ppm/cm: 0.42993

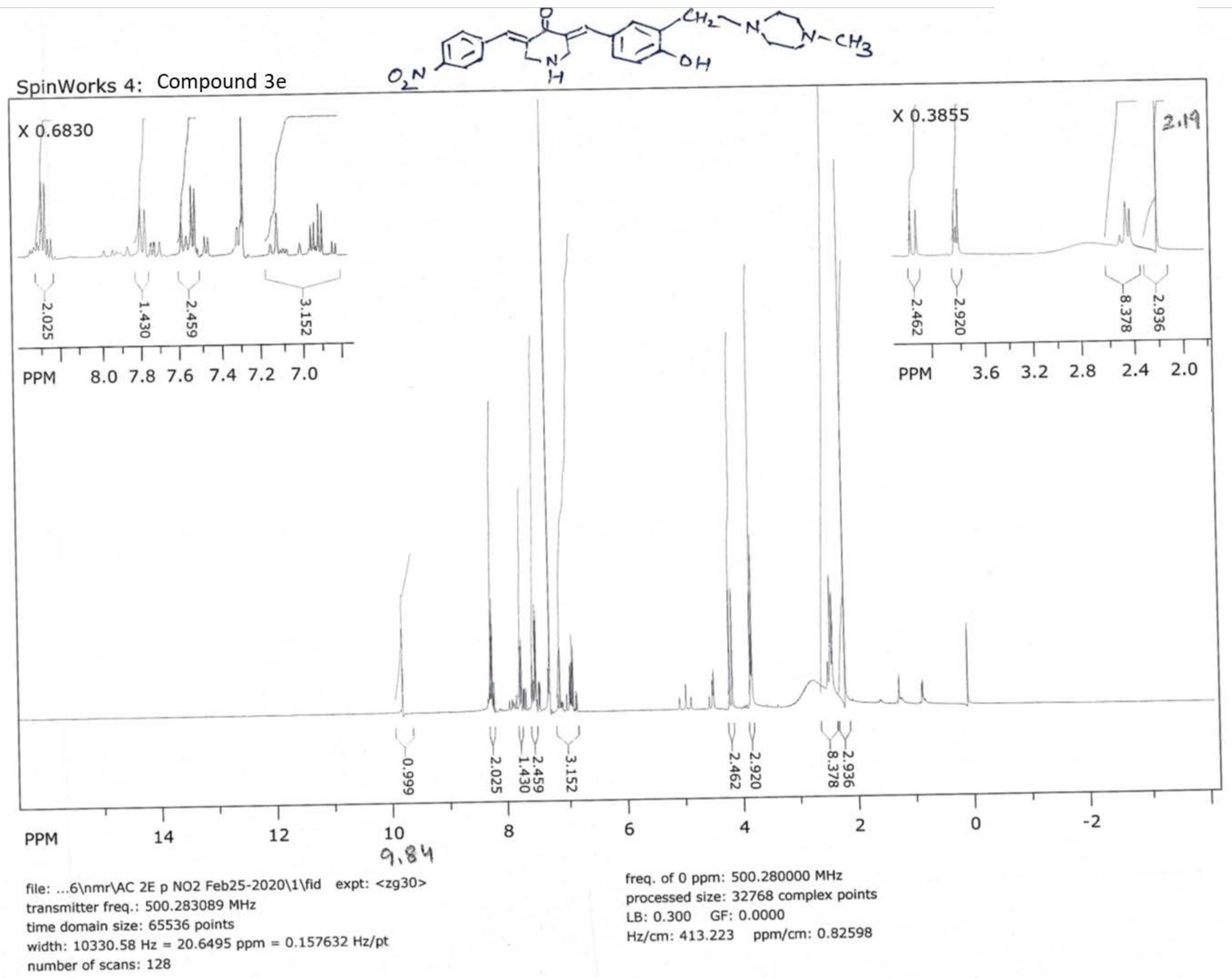
SpinWorks 4: Compound 3e
X 0.6830
X 0.3855
2.19
2.025
1.430
2.459
3.152
2.462
2.920
8.378
2.936
0.999
PPM 8.0 7.8 7.6 7.4 7.2 7.0
PPM 3.6 3.2 2.8 2.4 2.0
PPM 14 12 10 8 6 4 2 0 -2
9.84
file: ...6\nmr\AC 2E p NO2 Feb25-2020\1\fid expt: <zg30>
transmitter freq.: 500.283089 MHz
time domain size: 65536 points
width: 10330.58 Hz = 20.6495 ppm = 0.157632 Hz/pt
number of scans: 128
freq. of 0 ppm: 500.280000 MHz
processed size: 32768 complex points
LB: 0.300 GF: 0.0000
Hz/cm: 413.223 ppm/cm: 0.82598

Spectra S2. ^{13}C NMR spectra of compounds **2a**, **2b**, **2c**, **2d**, **3a**, **3b**, **3d**, and **3e**.

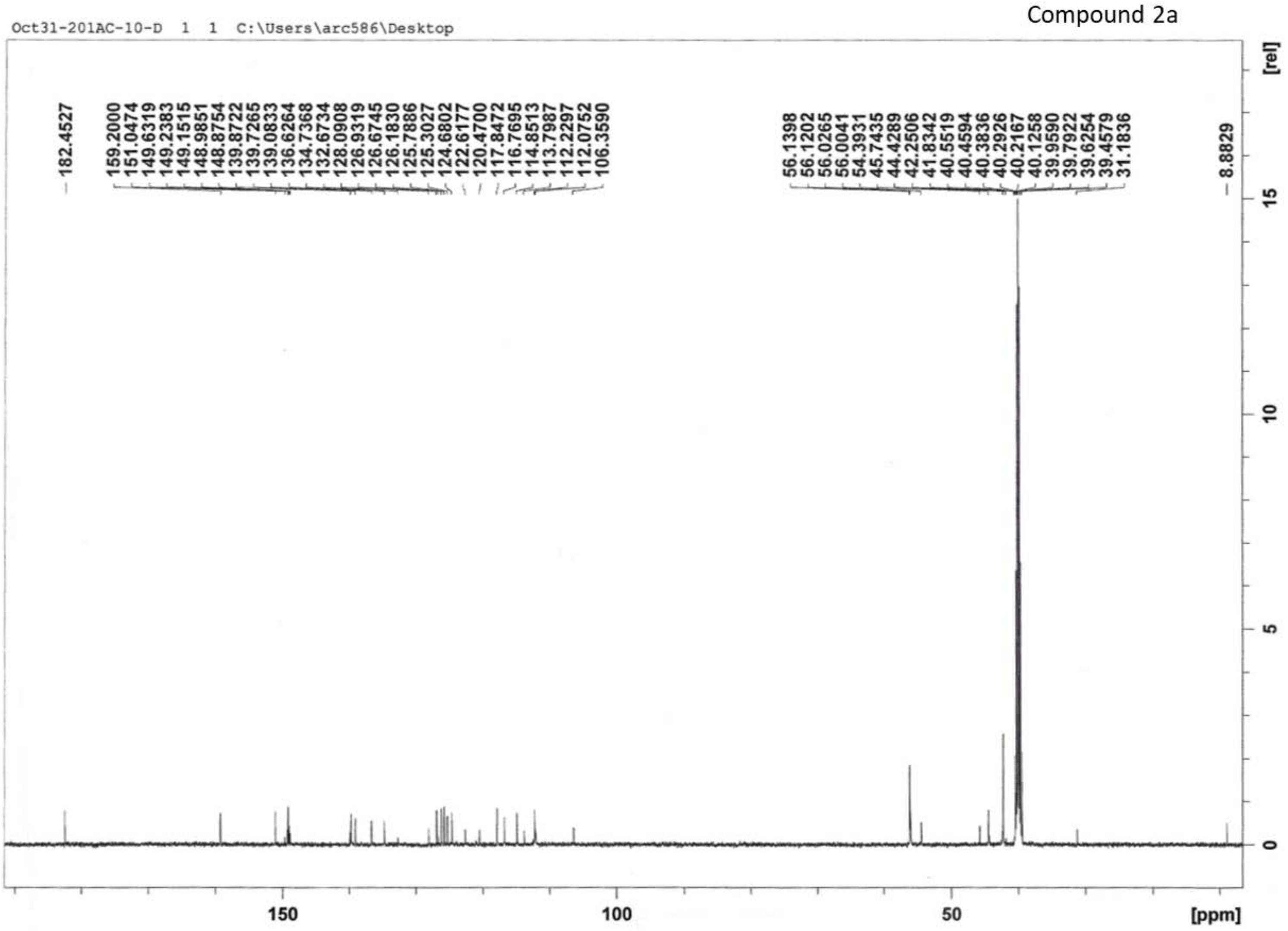

Compound 2b

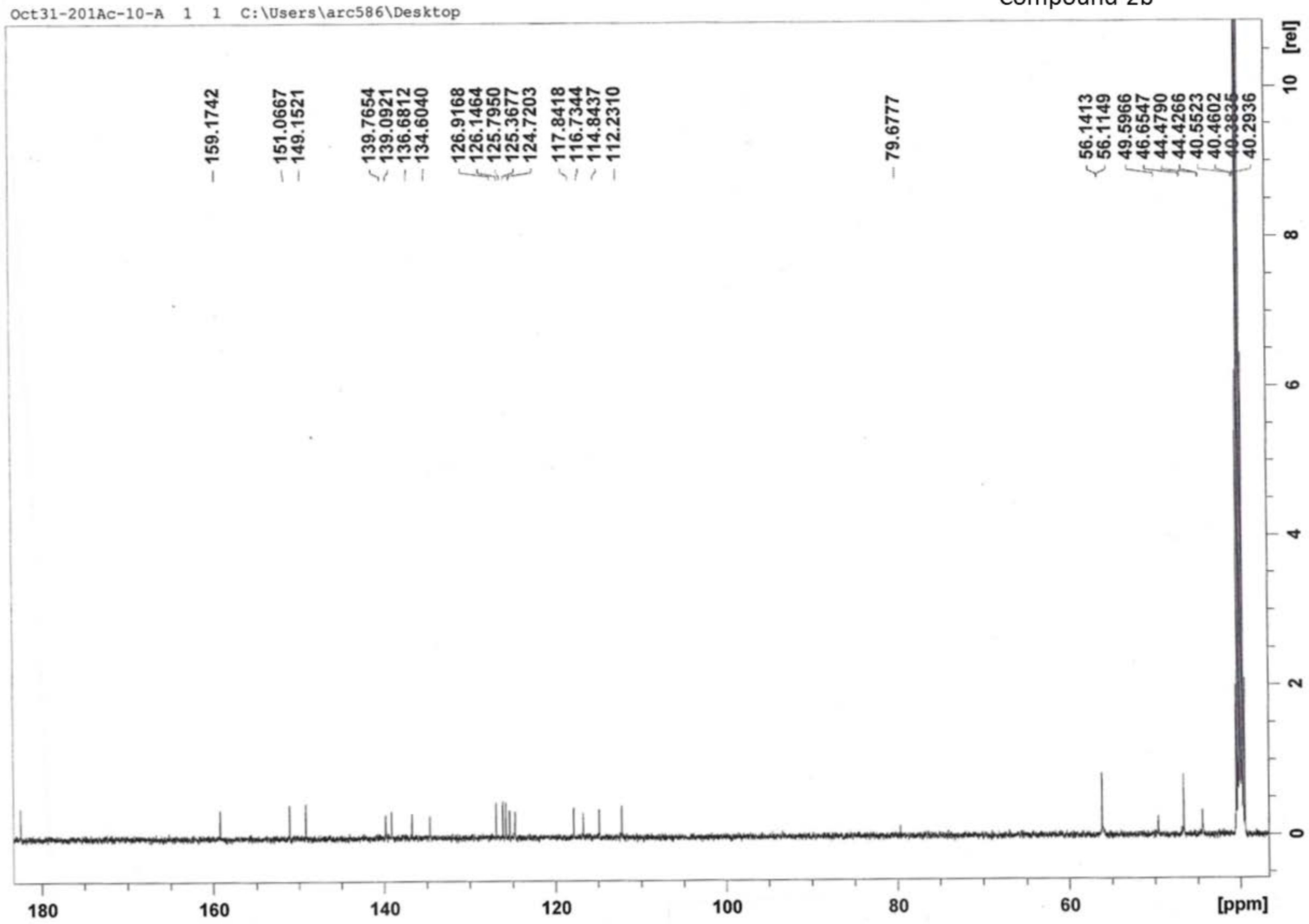

Oct31-201Ac-10-A 1 1 C:\Users\arc586\Desktop
159.1742
151.0667
149.1521
139.7654
139.0921
136.6812
134.6040
126.9168
126.1464
125.7950
125.3677
124.7203
117.8418
116.7344
114.8437
112.2310
79.6777
56.1413
56.1149
49.5966
46.6547
44.4790
44.4266
40.5523
40.4602
40.3835
40.2936
[rel]
10
8
6
4
2
0
180
160
140
120
100
80
60
[ppm]

Compound 2c

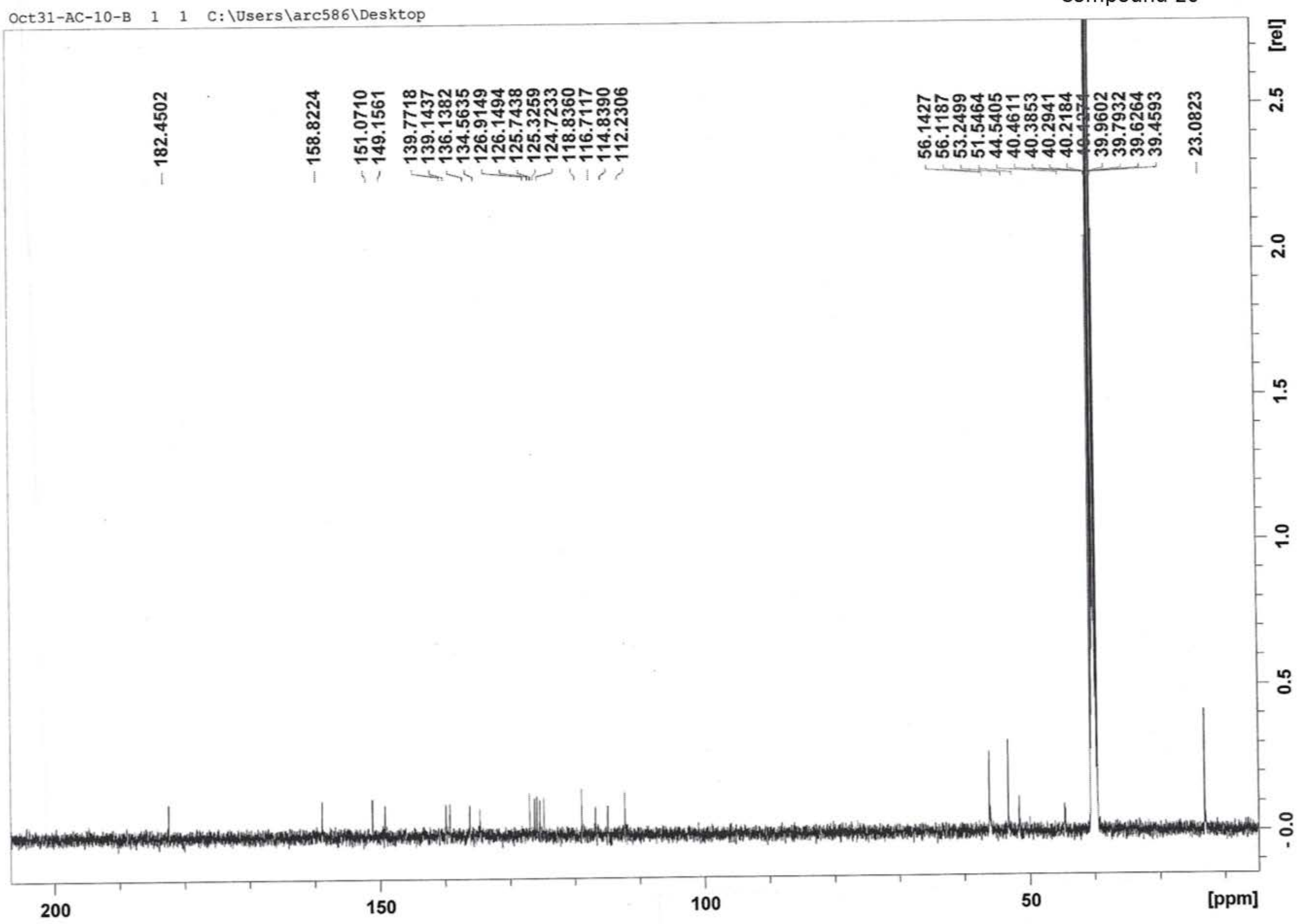
Oct31-AC-10-B 1 1 C:\Users\arc586\Desktop
182.4502
158.8224
151.0710
149.1561
139.7718
139.1437
136.1382
134.5635
126.9149
126.1494
125.7438
125.3259
124.7233
118.8360
116.7117
114.8390
112.2306
56.1427
56.1187
53.2499
51.5464
44.5405
40.4611
40.3853
40.2941
40.2184
39.9602
39.7932
39.6264
39.4593
23.0823
[rel]
2.5
2.0
1.5
1.0
0.5
-0.0
200
150
100
50
[ppm]

Compound 2d

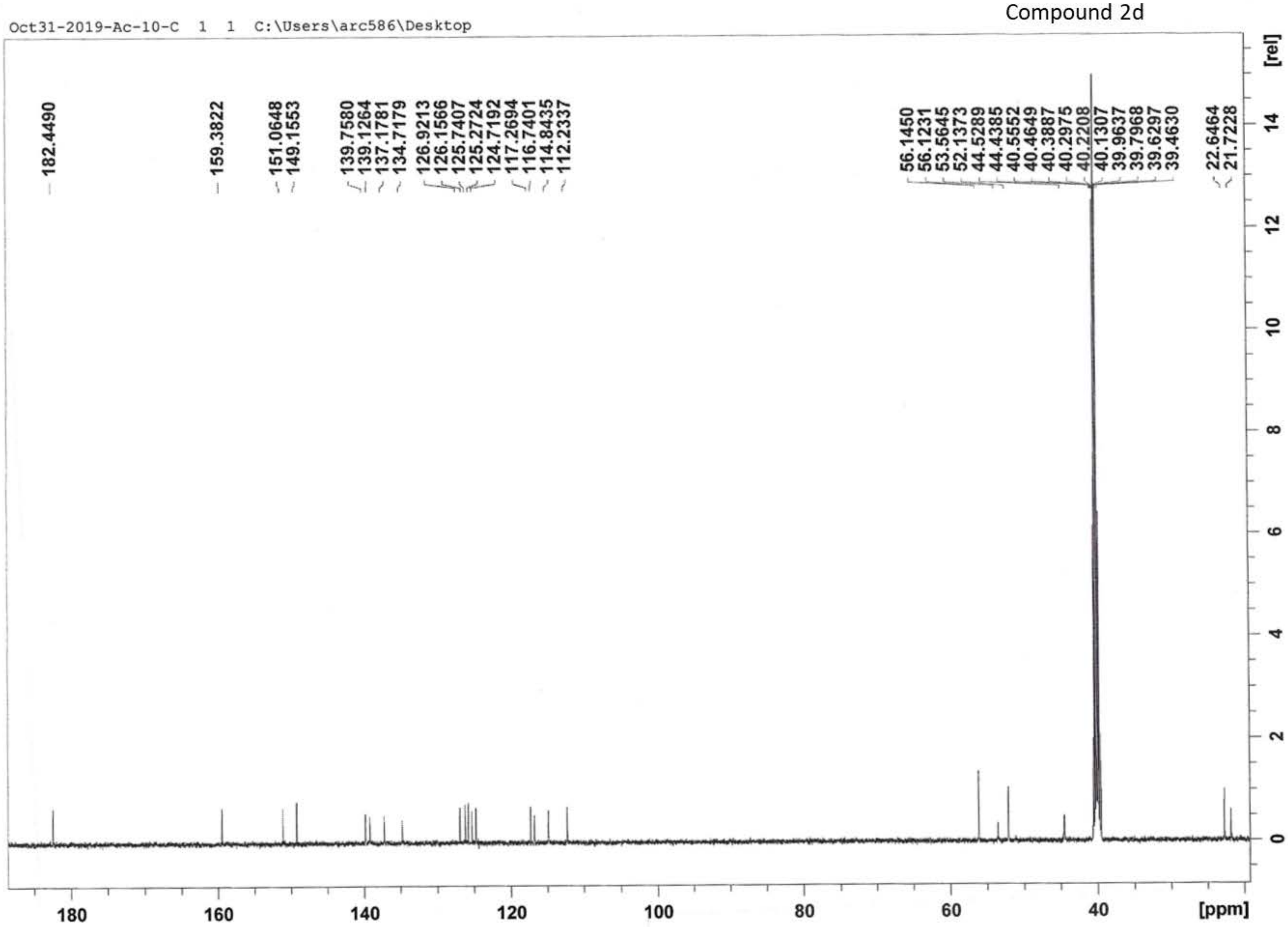
Oct31-2019-Ac-10-C 1 1 C:\Users\arc586\Desktop
182.4490
159.3822
151.0648
149.1553
139.7580
139.1264
137.1781
134.7179
126.9213
126.1566
125.7407
125.2724
124.7192
117.2694
116.7401
114.8435
112.2337
56.1450
56.1231
53.5645
52.1373
44.5289
44.4385
40.5552
40.4649
40.3887
40.2975
40.2208
40.1307
39.9637
39.7968
39.6297
39.4630
22.6464
21.7228
[rel]
[ppm]

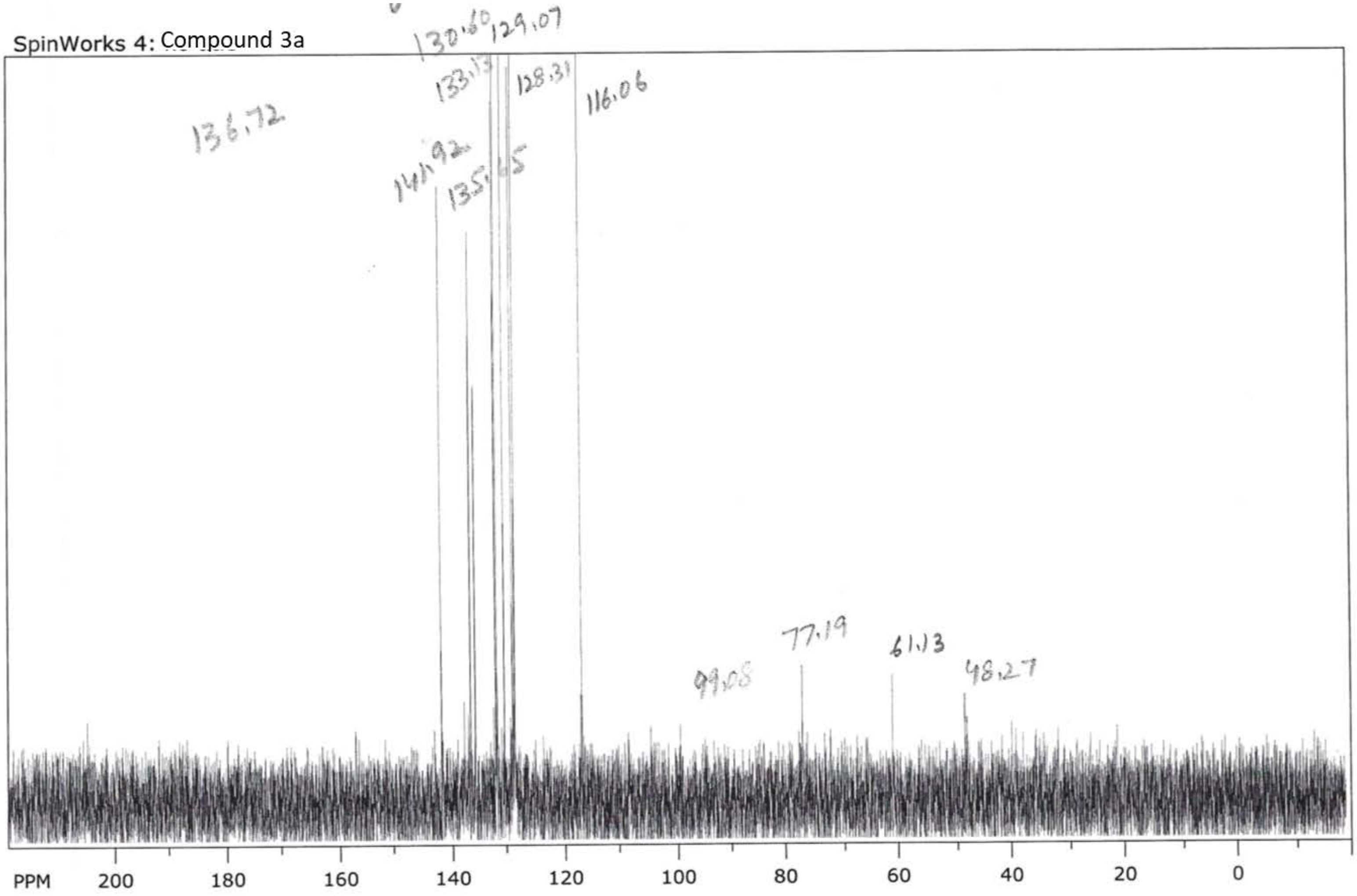

file: ...nzaldehyde deriv. Jan29-2020\2\fid expt: <dept90>
transmitter freq.: 125.808086 MHz
time domain size: 65536 points
width: 30030.03 Hz = 238.6971 ppm = 0.458222 Hz/pt
number of scans: 256

freq. of 0 ppm: 125.795506 MHz
processed size: 32768 complex points
LB: 1.000 GF: 0.0000
Hz/cm: 1201.201 ppm/cm: 9.54789

SpinWorks 4: Compound 3b

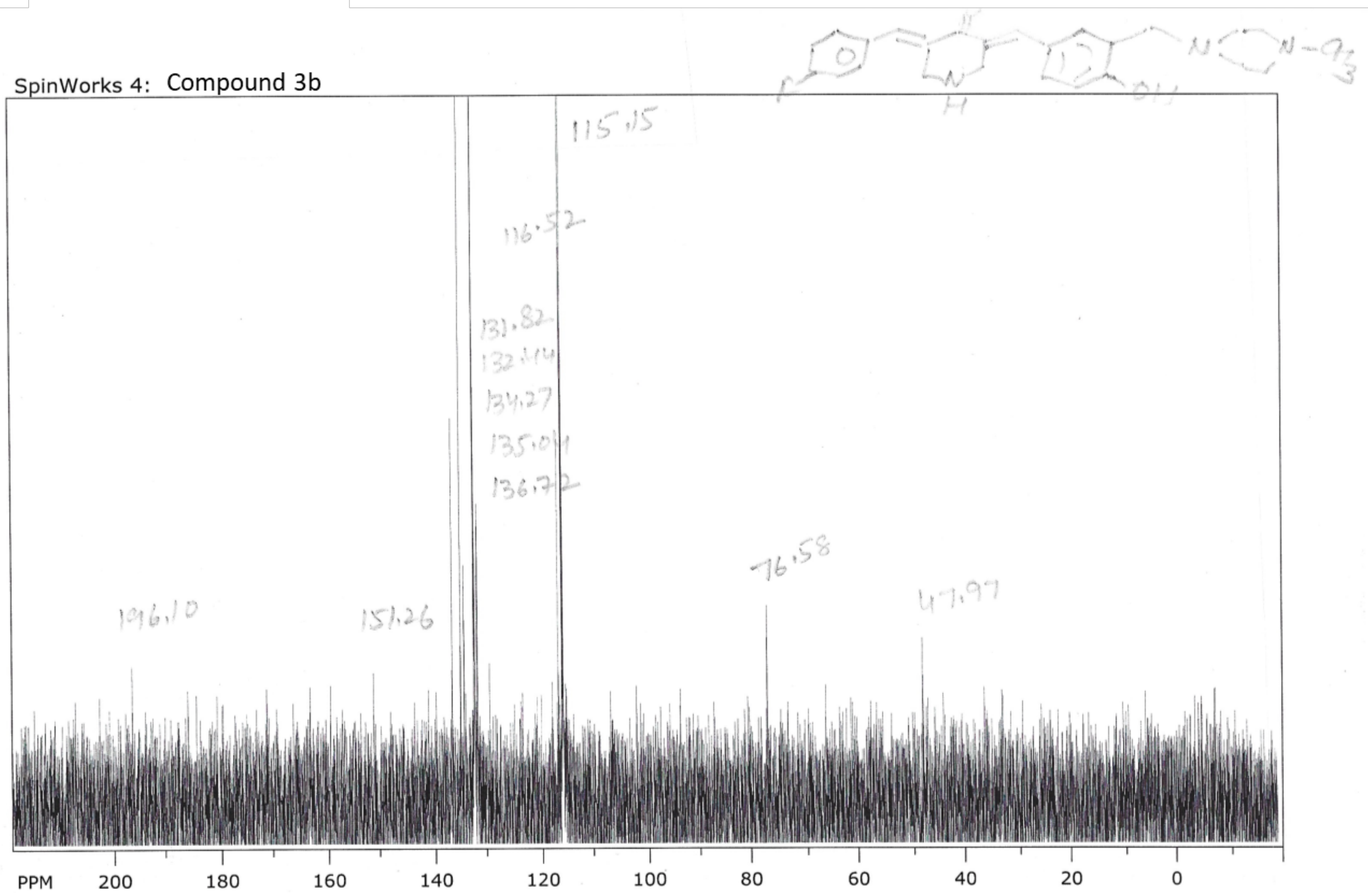

file: ...ta\u\fbr2\arc586\nmr\2699 AC\2\fid expt: <deptsp90>
transmitter freq.: 125.808086 MHz
time domain size: 65536 points
width: 30030.03 Hz = 238.6971 ppm = 0.458222 Hz/pt
number of scans: 256

freq. of 0 ppm: 125.795506 MHz
processed size: 32768 complex points
LB: 1.000 GF: 0.0000
Hz/cm: 1201.201 ppm/cm: 9.54789

SpinWorks 4: Compound 3d

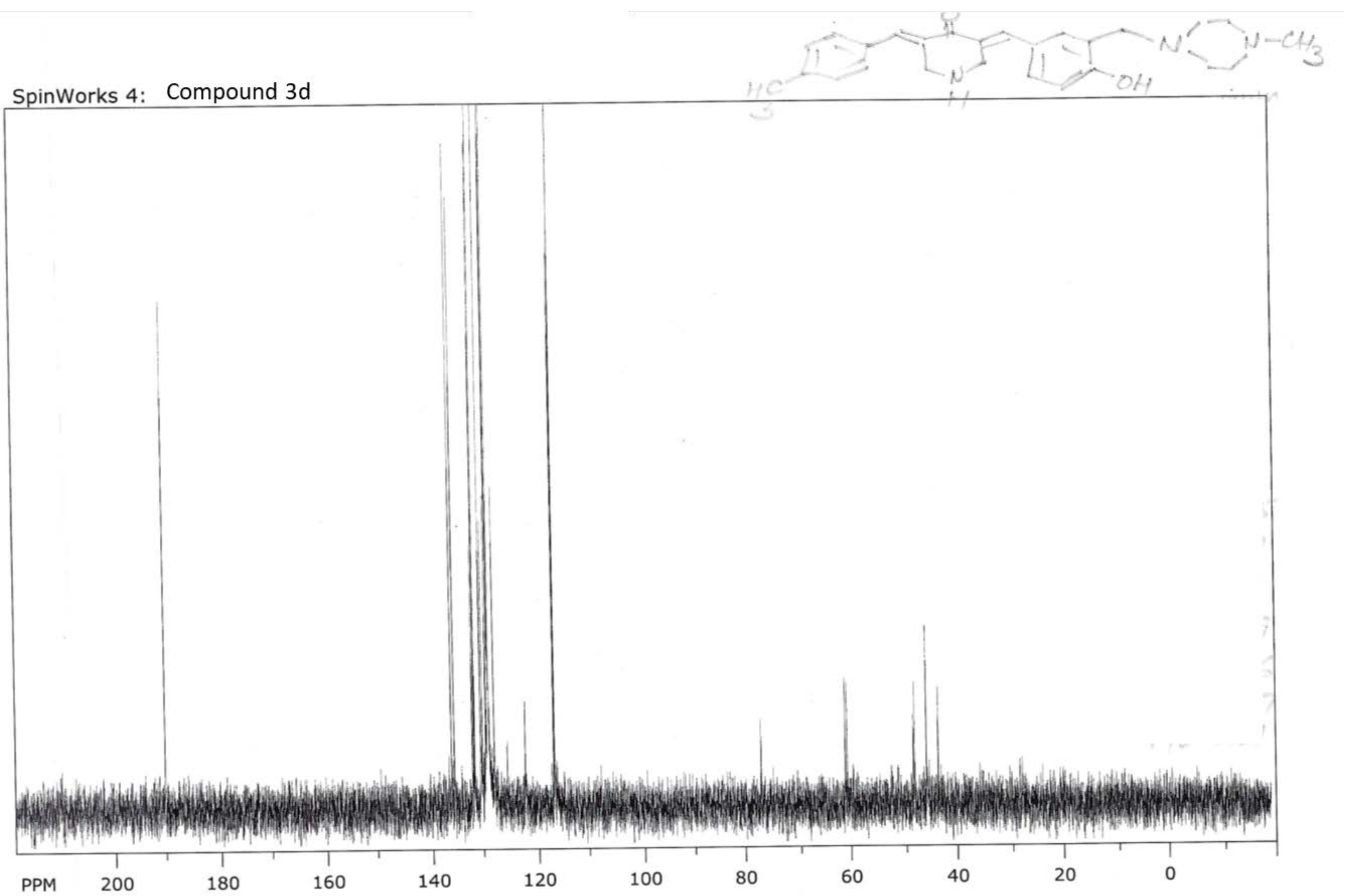

file: ...\u\fbr2\arc586\nmr\2701 AC 1\1\fid expt: <deptsp90>
transmitter freq.: 125.808086 MHz
time domain size: 65536 points
width: 30030.03 Hz = 238.6971 ppm = 0.458222 Hz/pt
number of scans: 256

freq. of 0 ppm: 125.795506 MHz
processed size: 32768 complex points
LB: 1.000 GF: 0.0000
Hz/cm: 1201.201 ppm/cm: 9.54789

SpinWorks 4: Compound 3e

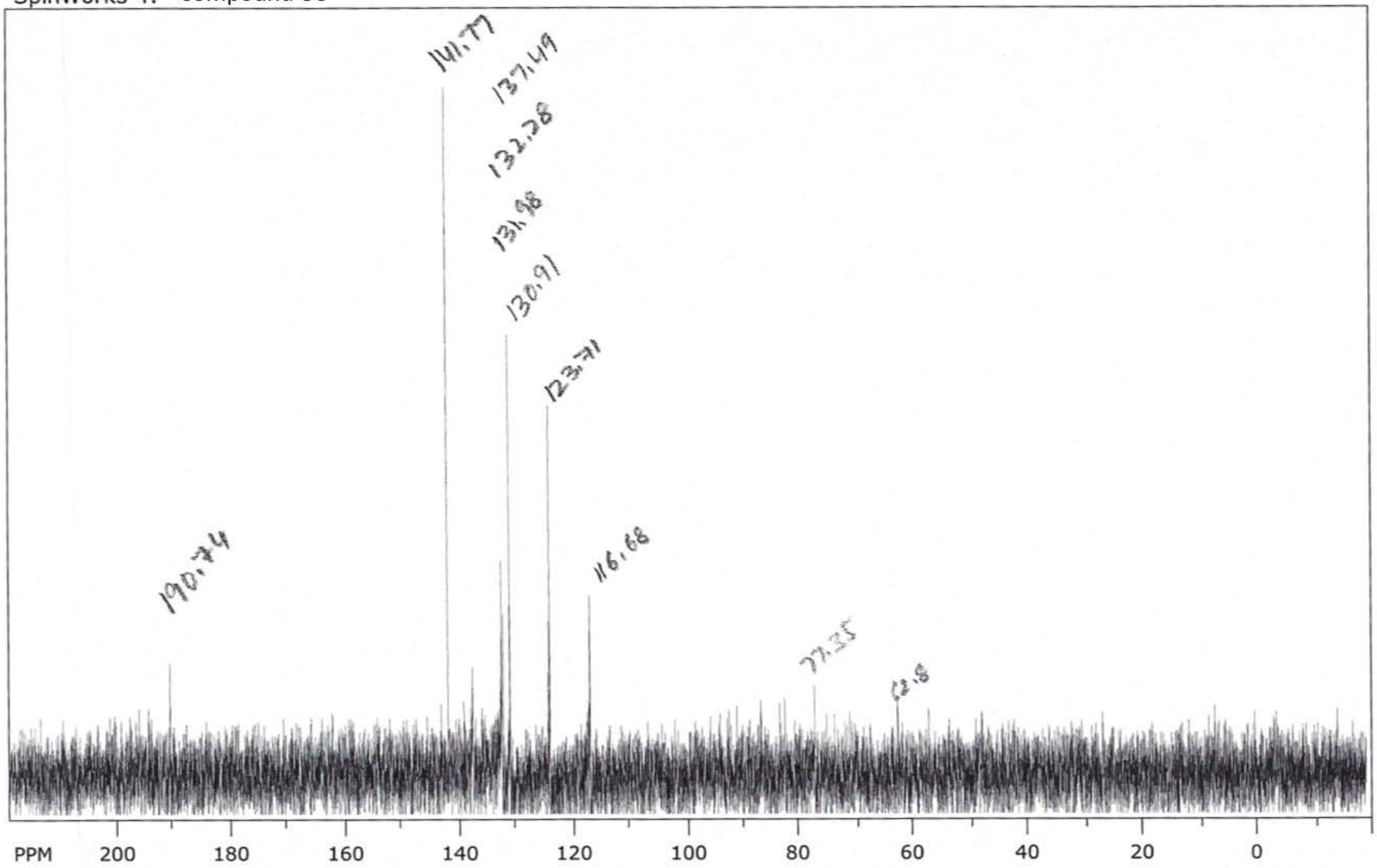

file: ...6\nmr\AC 2E p NO2 Feb25-2020\2\fid expt: <dept90>
transmitter freq.: 125.808086 MHz
time domain size: 65536 points
width: 30030.03 Hz = 238.6971 ppm = 0.458222 Hz/pt
number of scans: 256

freq. of 0 ppm: 125.795506 MHz
processed size: 32768 complex points
LB: 1.000 GF: 0.0000
Hz/cm: 1201.201 ppm/cm: 9.54789

Spectra S3. Mass spectra of compounds **2a**-**f** and **3a**-**e**.

Compound 2a

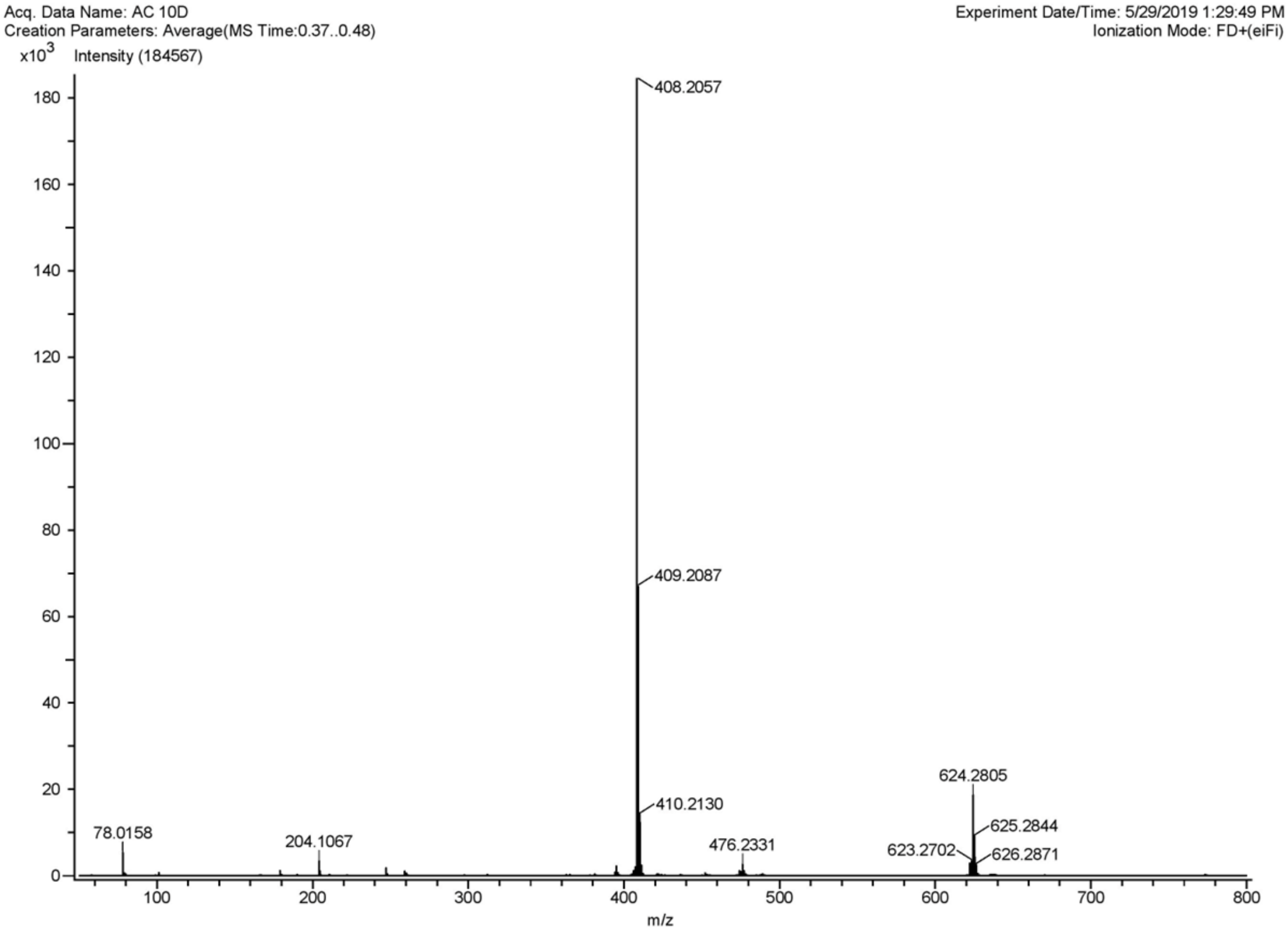

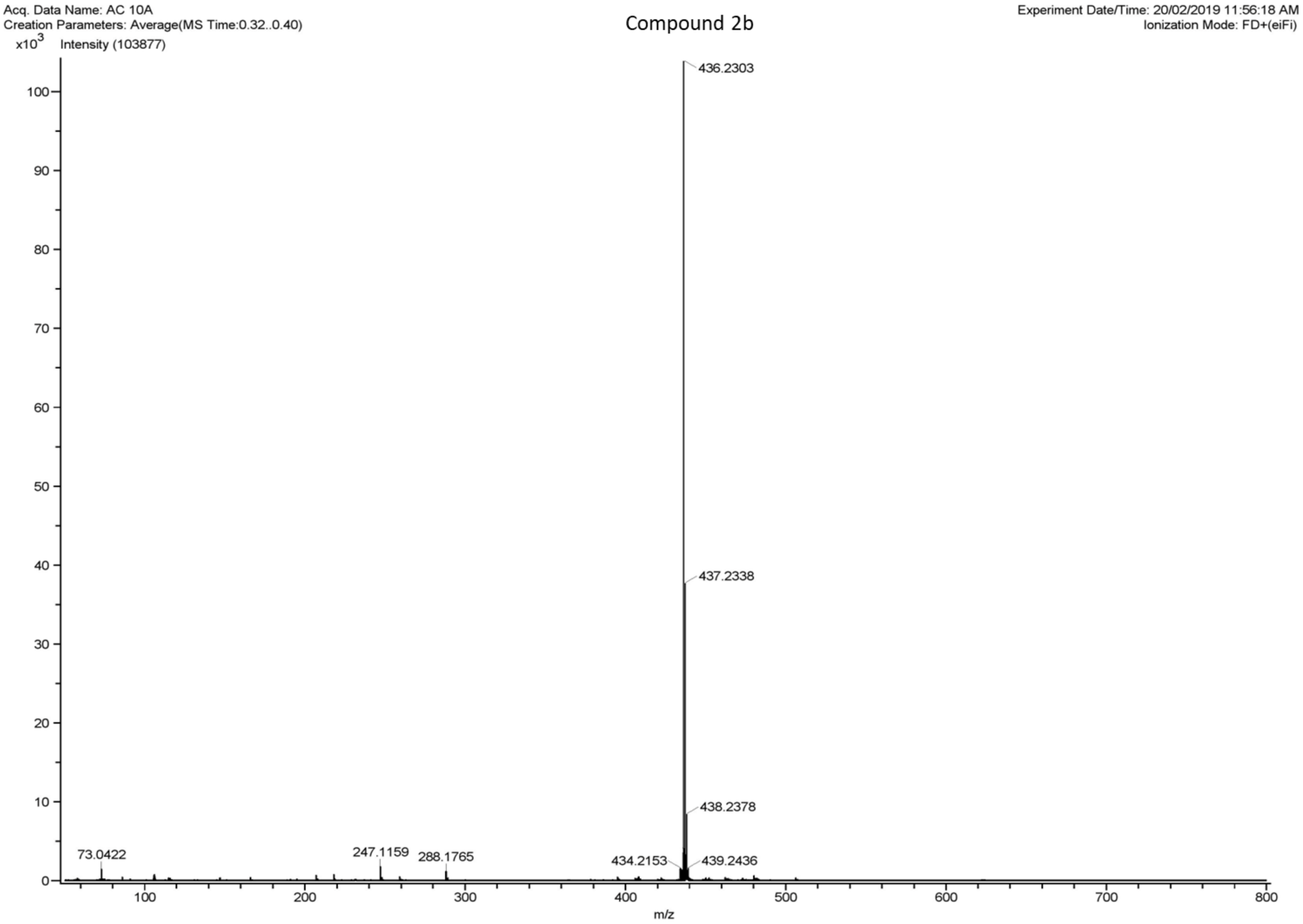
Acq. Data Name: AC 10A
Creation Parameters: Average(MS Time:0.32..0.40)
x10^3 Intensity (103877)
Compound 2b
Experiment Date/Time: 20/02/2019 11:56:18 AM
Ionization Mode: FD+(eiFi)
436.2303
437.2338
438.2378
439.2436
434.2153
288.1765
247.1159
73.0422
m/z

Compound 2c

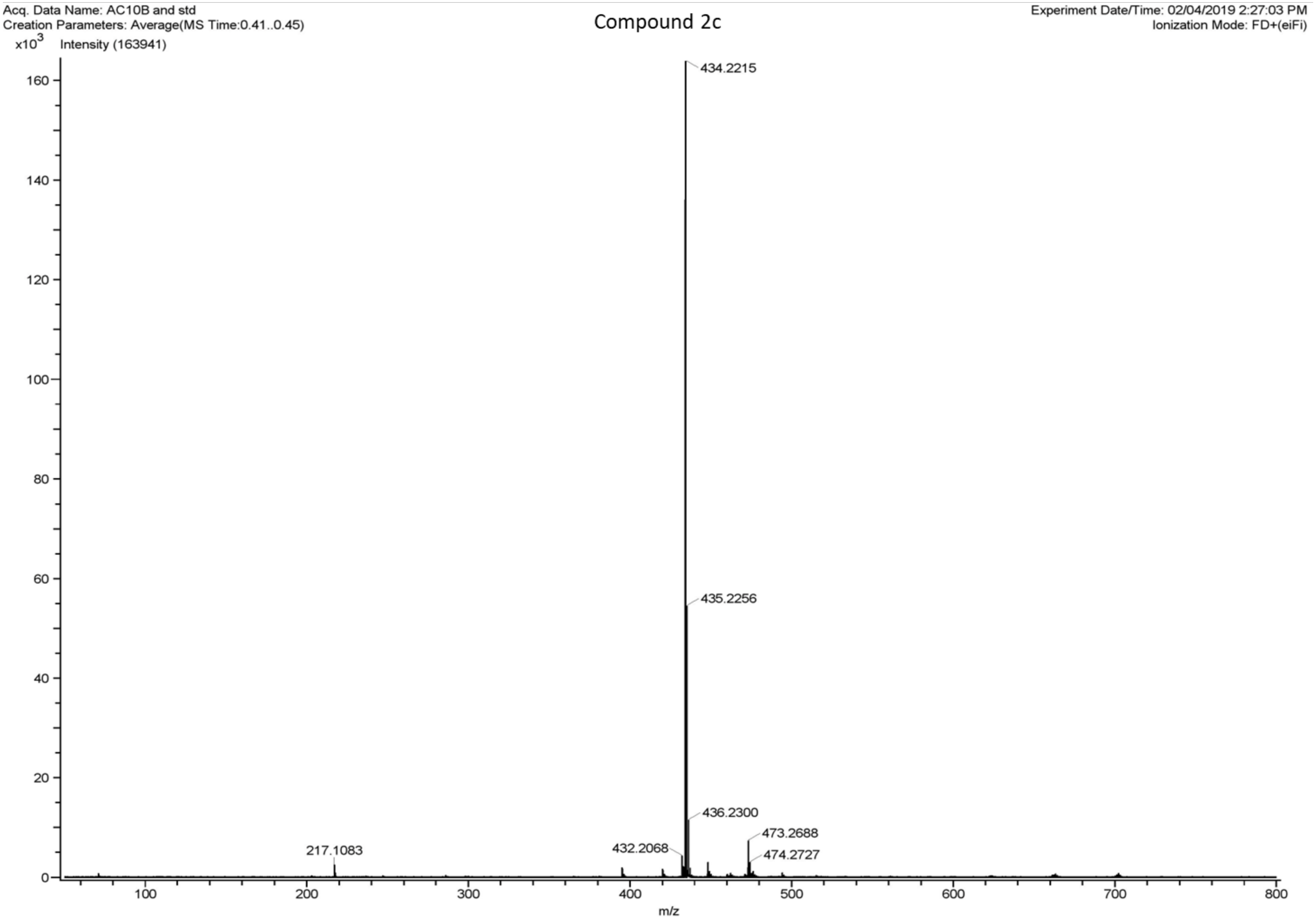

Acq. Data Name: AC10B and std
Creation Parameters: Average(MS Time:0.41..0.45)
Experiment Date/Time: 02/04/2019 2:27:03 PM
Ionization Mode: FD+(eiFi)
x10^3 Intensity (163941)
434.2215
435.2256
436.2300
432.2068
473.2688
474.2727
217.1083
m/z

Compound 2d

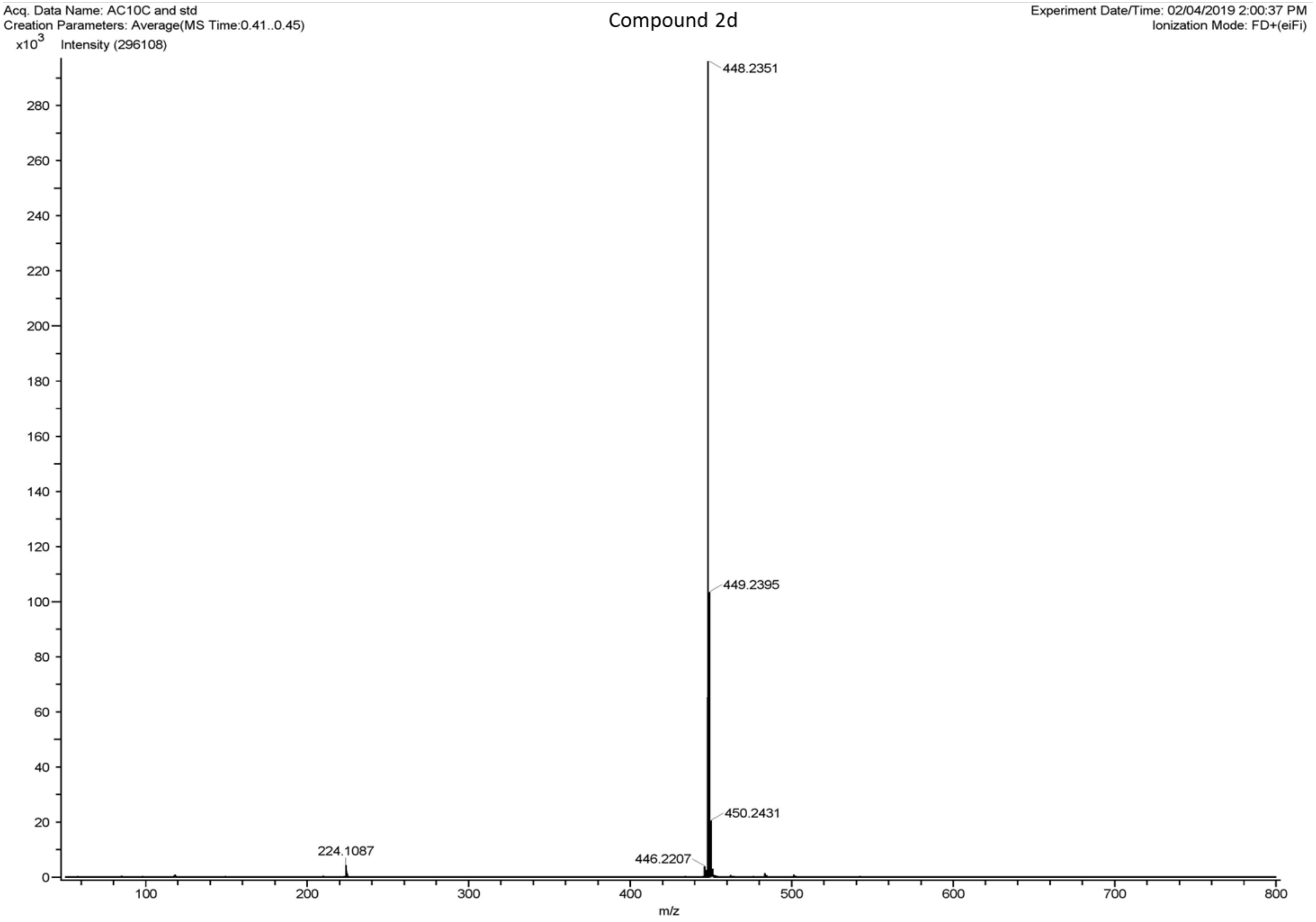

Acq. Data Name: AC10C and std
Creation Parameters: Average(MS Time:0.41..0.45)
Experiment Date/Time: 02/04/2019 2:00:37 PM
Ionization Mode: FD+(eiFi)
x10^3 Intensity (296108)
448.2351
449.2395
450.2431
446.2207
224.1087
m/z

Compound 2e

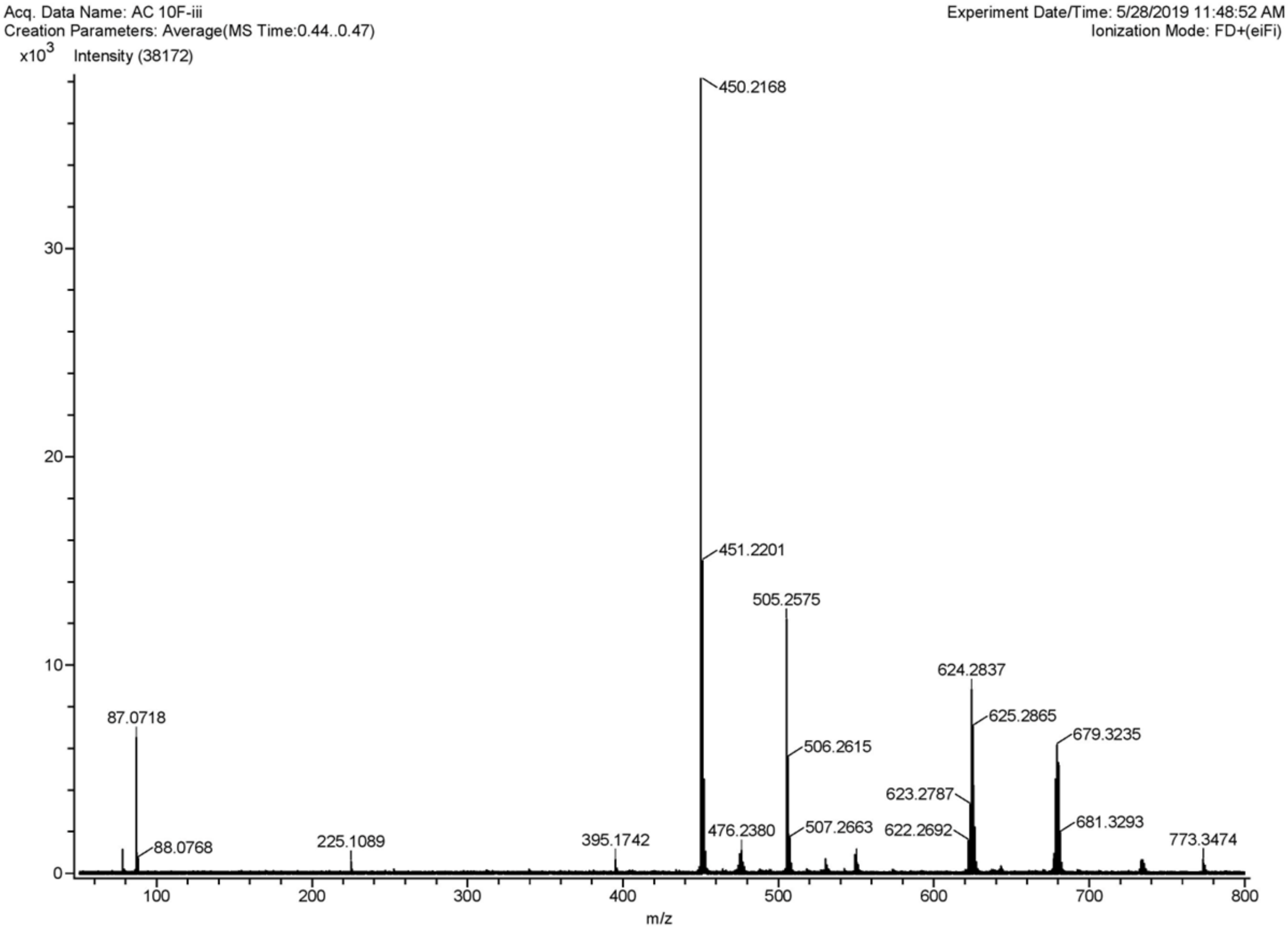

Acq. Data Name: AC 10F-iii
Creation Parameters: Average(MS Time:0.44..0.47)
Experiment Date/Time: 5/28/2019 11:48:52 AM
Ionization Mode: FD+(eiFi)
x10^3 Intensity (38172)
450.2168
451.2201
505.2575
624.2837
625.2865
679.3235
506.2615
623.2787
681.3293
87.0718
88.0768
225.1089
395.1742
476.2380
507.2663
622.2692
773.3474
m/z

Compound 2f

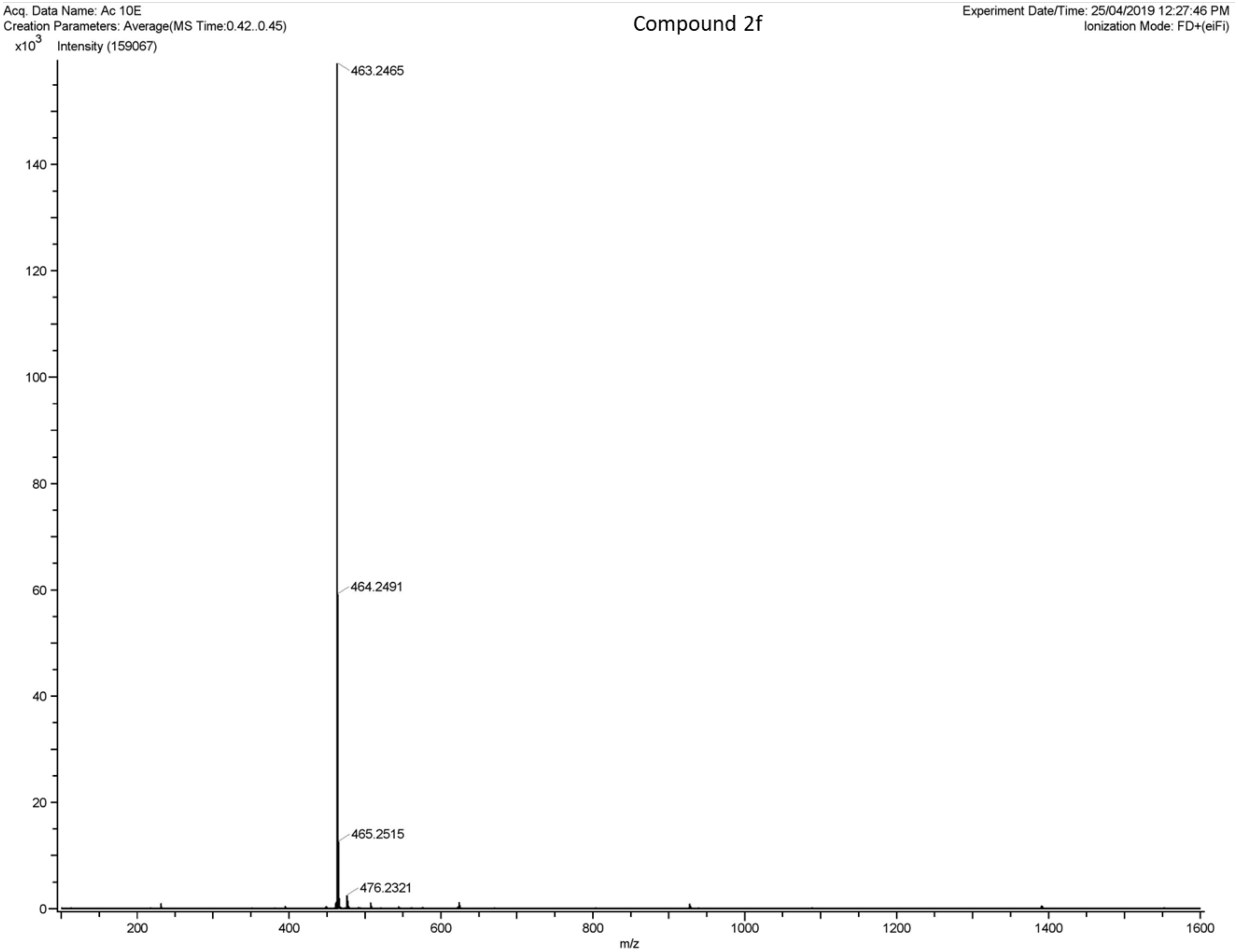
Acq. Data Name: Ac 10E
Creation Parameters: Average(MS Time:0.42..0.45)
Experiment Date/Time: 25/04/2019 12:27:46 PM
Ionization Mode: FD+(eiFi)
x10^3 Intensity (159067)
463.2465
464.2491
465.2515
476.2321
m/z

Compound 3a

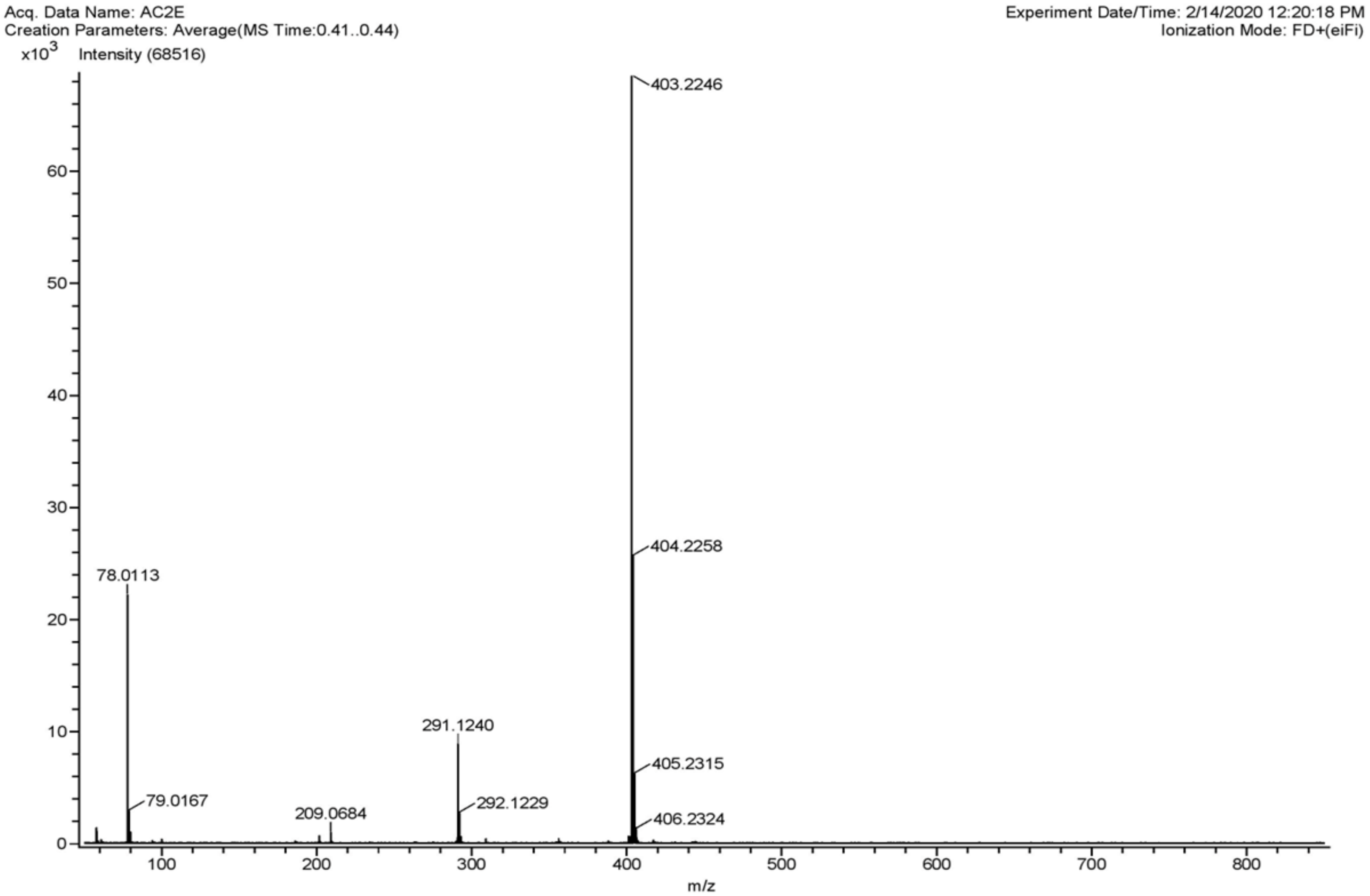

Acq. Data Name: AC2E
Creation Parameters: Average(MS Time:0.41..0.44)
Experiment Date/Time: 2/14/2020 12:20:18 PM
Ionization Mode: FD+(eiFi)
x10^3 Intensity (68516)
403.2246
404.2258
405.2315
406.2324
78.0113
79.0167
209.0684
291.1240
292.1229
m/z

Compound 3b

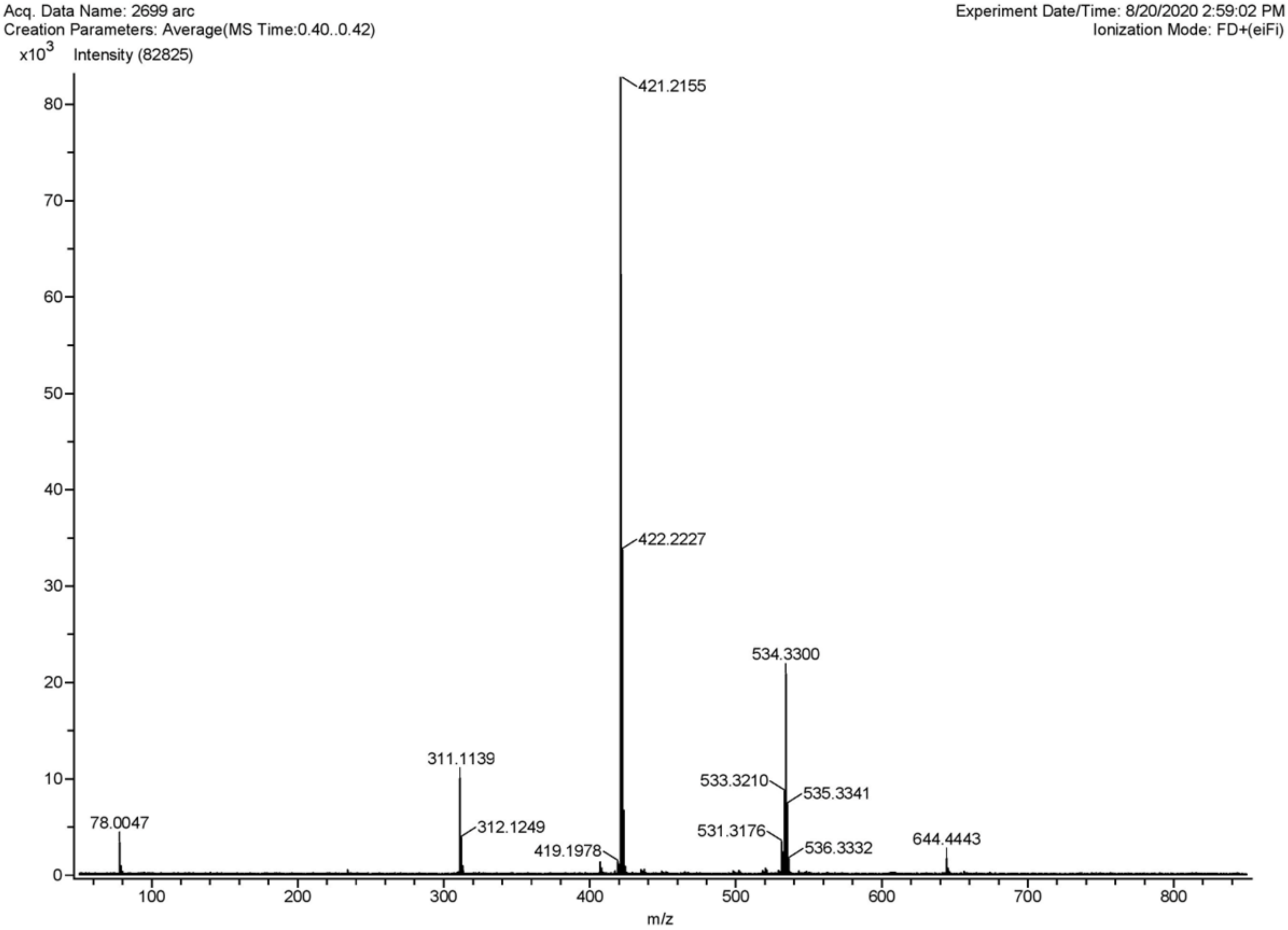

Acq. Data Name: 2699 arc
Creation Parameters: Average(MS Time:0.40..0.42)
x10^3 Intensity (82825)
Experiment Date/Time: 8/20/2020 2:59:02 PM
Ionization Mode: FD+(eiFi)
421.2155
422.2227
534.3300
311.1139
533.3210
535.3341
78.0047
312.1249
531.3176
419.1978
536.3332
644.4443
0
10
20
30
40
50
60
70
80
100
200
300
400
500
600
700
800
m/z

Compound 3c

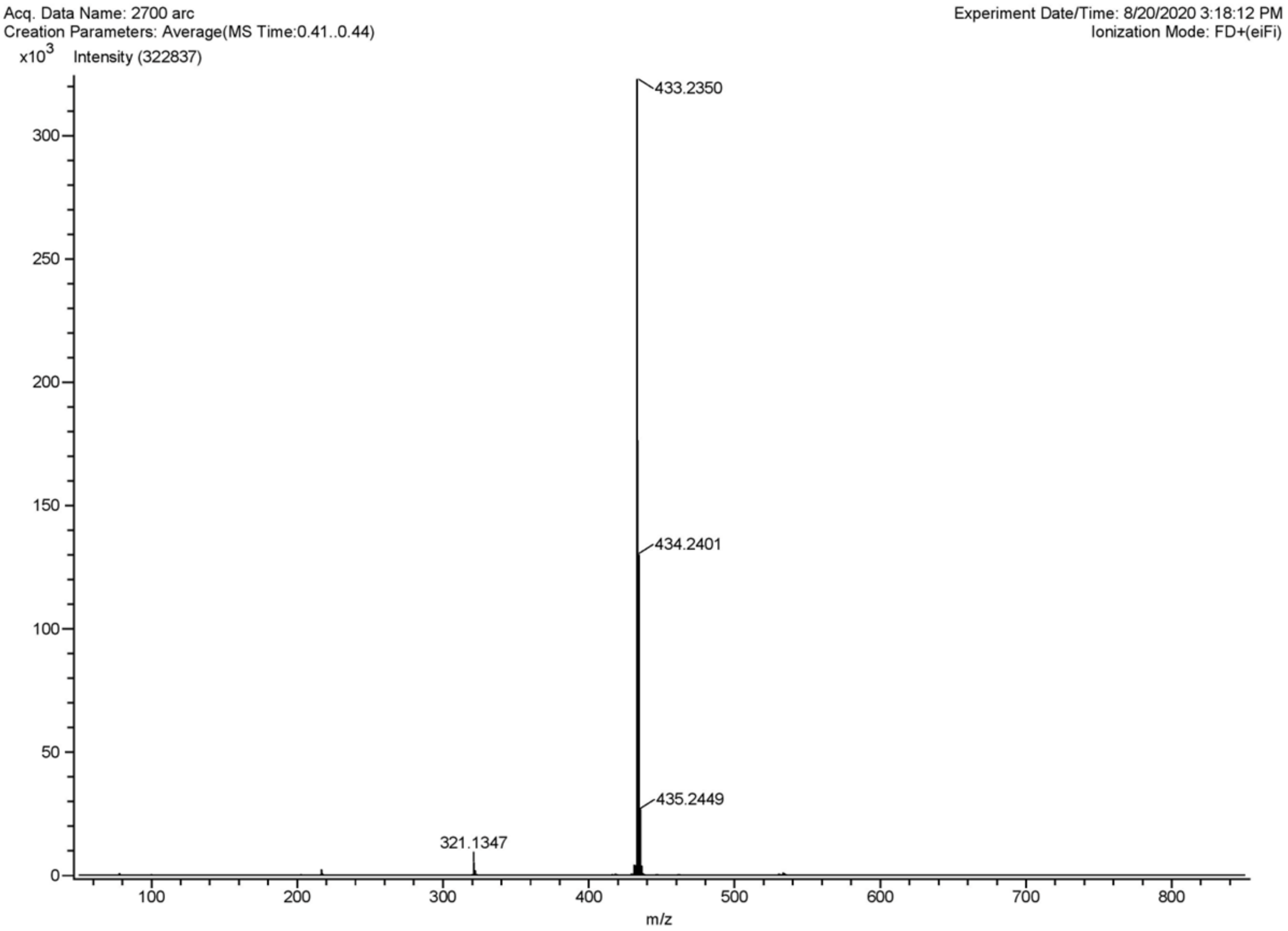

Acq. Data Name: 2700 arc
Creation Parameters: Average(MS Time:0.41..0.44)
Experiment Date/Time: 8/20/2020 3:18:12 PM
Ionization Mode: FD+(eiFi)
x10^3 Intensity (322837)
433.2350
434.2401
435.2449
321.1347
0
50
100
150
200
250
300
100
200
300
400
500
600
700
800
m/z

Compound 3d

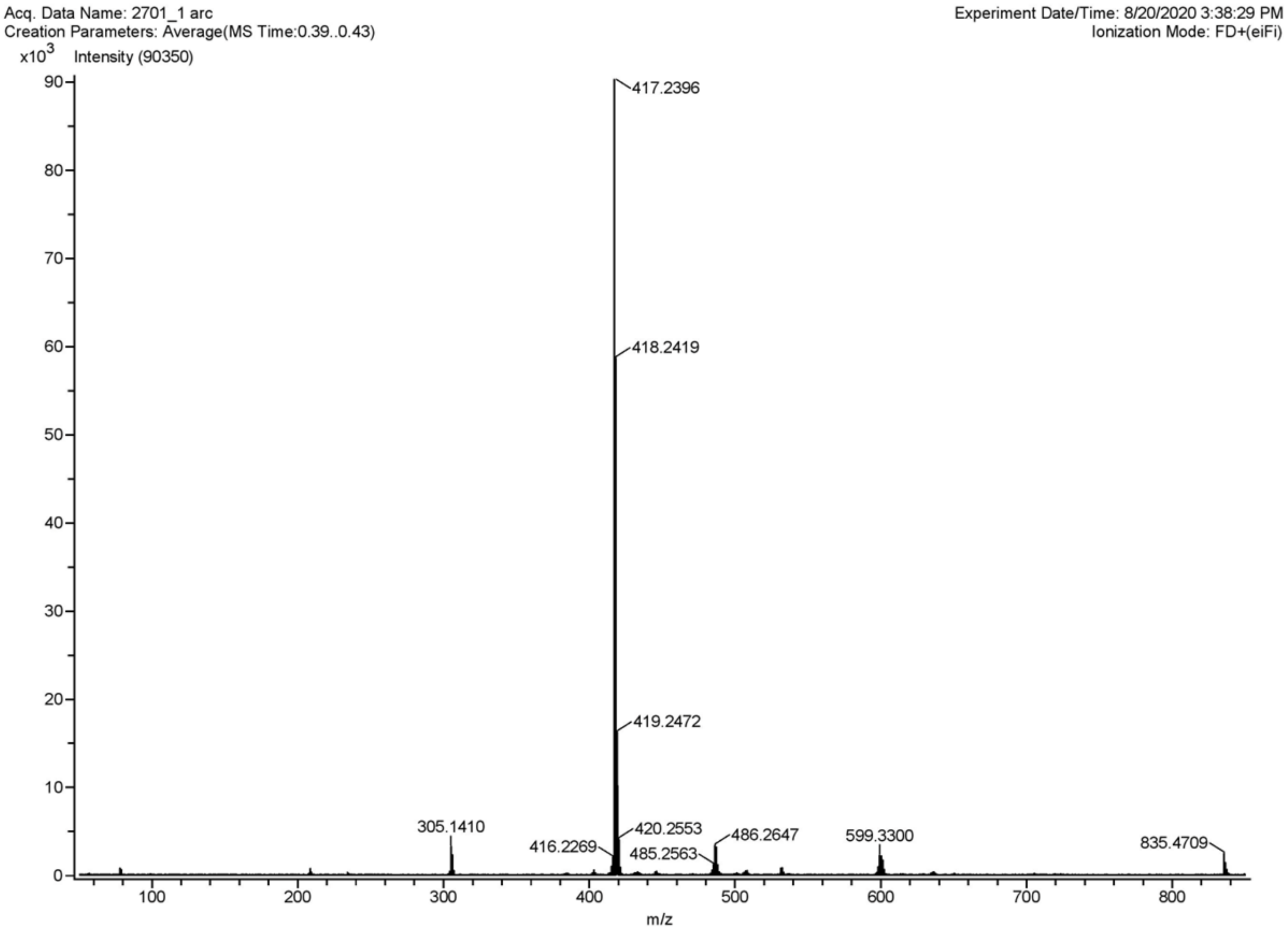

Acq. Data Name: 2701_1 arc
Creation Parameters: Average(MS Time:0.39..0.43)
Experiment Date/Time: 8/20/2020 3:38:29 PM
Ionization Mode: FD+(eiFi)
x10^3 Intensity (90350)
417.2396
418.2419
419.2472
305.1410
416.2269
420.2553
485.2563
486.2647
599.3300
835.4709
m/z

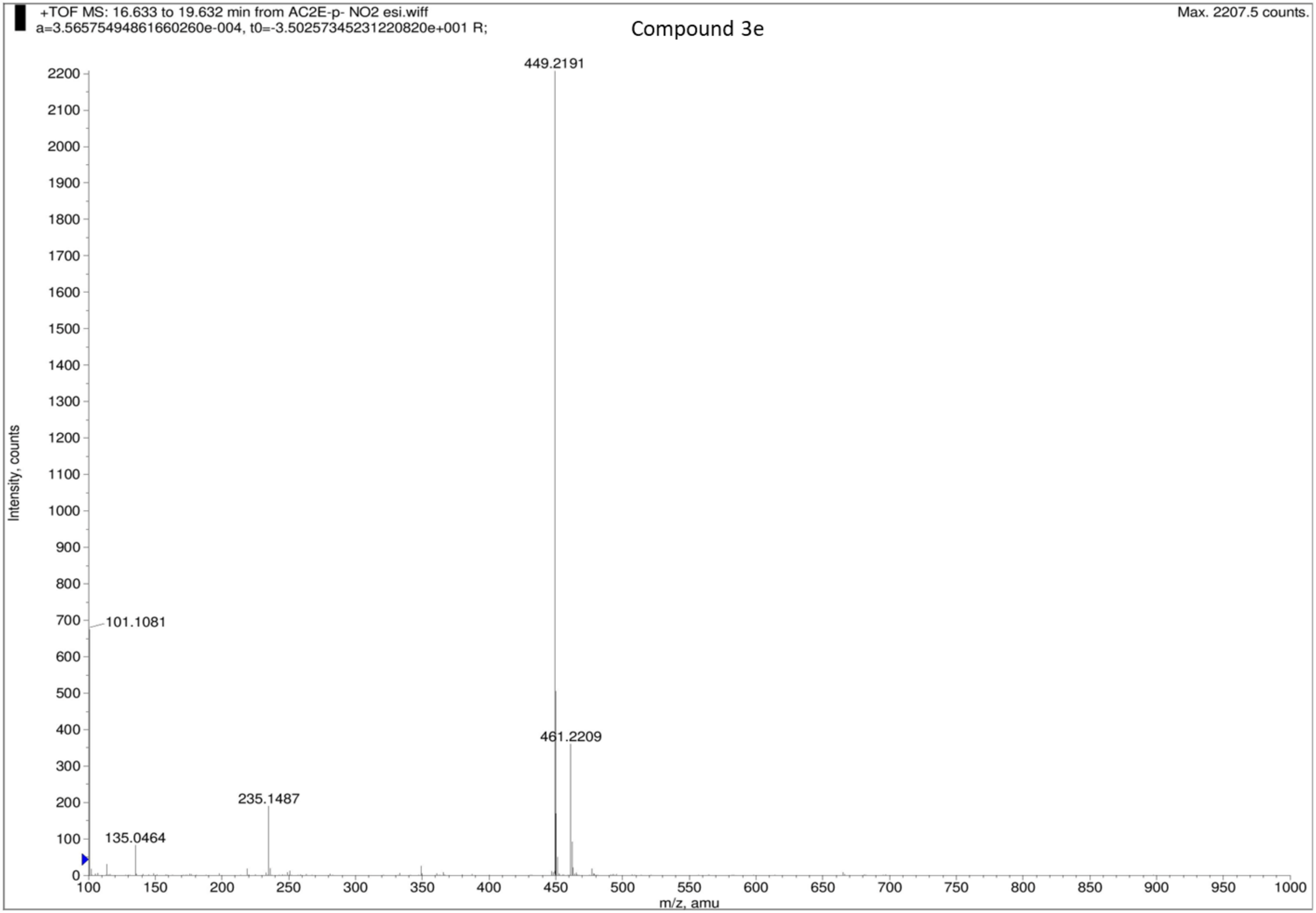
+TOF MS: 16.633 to 19.632 min from AC2E-p- NO2 esi.wiff
a=3.56575494861660260e-004, t0=-3.50257345231220820e+001 R;
Compound 3e
Max. 2207.5 counts.
449.2191
101.1081
461.2209
235.1487
135.0464
Intensity, counts
m/z, amu
